# The Omp85 family protein, TamA, exhibits characteristics of a suitable drug target against Pseudomonas aeruginosa

**DOI:** 10.1099/mic.0.001674

**Published:** 2026-04-21

**Authors:** Rachael Duodu, David J. Boocock, Lesley Hoyles, Jack C. Leo

**Affiliations:** 1 Antimcrobial Resistance, Omics and Microbiota Group, Centre for Systems Health and Integrated Metabolic Research, Department of Bioscience, School of Science and Technology, Nottingham Trent University, Nottingham, UK; 2 John van Geest Cancer Research Centre, Nottingham Trent University, Nottingham, UK

**Keywords:** outer membrane proteins, proteomics, transcriptomics, translocation and assembly module

## Abstract

The outer membrane (OM) of Gram-negative bacteria is crucial for cell stability and virulence and acts as a permeability barrier. The biogenesis, assembly and regulation of proteins in the OM are, therefore, attractive areas of study that could lead to identifying novel drug targets. The translocation and assembly module (TAM), composed of TamA and TamB, facilitates the insertion of some *β*-barrel proteins into the OM of *Escherichia coli* and *Klebsiella pneumoniae* and has also been implicated in lipid homeostasis. However, its role in *Pseudomonas aeruginosa* remains mostly uncharacterized. To investigate the TAM’s function and drug target potential in *P. aeruginosa*, we generated both single- and double-gene TAM knockouts and assessed their fitness using competition growth assays against WT strains. The WT significantly outcompeted the TAM mutants, indicating a fitness defect. Proteomic analysis revealed surprisingly similar profiles between WT and the double-knockout strains, while single-knockout strains showed changes in OM proteins and reduced expression of flagellar components consistent with attenuated swimming motility observed in ∆*tamA*. Single-knockout mutants exhibited differential levels of expression of lipoproteins of the *β*-barrel assembly machinery, suggesting compensatory OM remodelling. *In vivo* infection assays using *Galleria mellonella* larvae demonstrated significantly higher survival rates when infected with TAM mutants, with *tamA* mutants showing the greatest attenuation in virulence. Our findings demonstrate a role the TAM plays in *P. aeruginosa* virulence and identify TamA as a potential drug target for the development of new antimicrobial therapies against *P. aeruginosa*.

## Data Summary

The RNA-seq data reported in this article are available from ArrayExpress under accession E-MTAB 15348. Individual sample accession numbers and their corresponding names used in this study are provided in Table S7. The mass spectrometry proteomics data have been deposited to the ProteomeXchange Consortium with the dataset identifier PXD067041. Supplementary material is available with the online version of this article, available through Figshare at https://doi.org/10.6084/m9.figshare.30902561 [1].

## Introduction

The outer membrane (OM) of Gram-negative bacteria provides mechanical protection and serves as a permeability barrier due to its asymmetry, consisting of phospholipids on its inside and lipopolysaccharides (LPSs) on its exterior [2]. It boasts abundant integral OM proteins (OMPs), most of which adopt a *β*-barrel structure [3].

The biogenesis of the OM involves the transport of precursors of its constituents from the cytoplasm, where they are synthesized, followed by insertion into the OM [4]. Nascent OMPs are transported into the periplasm through the Sec machinery. Periplasmic chaperones like SurA and Skp then escort unfolded OMPs [5] to the *β*-barrel assembly machinery (BAM), which mediates their correct folding into the OM. OMPs perform many important functions, including transport, virulence and bacterial survival, and, therefore, are good drug targets [3].

The BAM has been extensively studied during the past decade, and it is required in the assembly of most OMPs, including OmpA [6], OmpC [7], OmpF [8] and OmpT [9], Ag43 [10], intimin [11], TolC [12] and PhoE [13] in *Escherichia coli*, among many others. The BAM consists of BamA, an Omp85 family integral membrane protein [14,15] connected by its five polypeptide transport-associated motif (POTRA) domains to four lipoproteins, BamB, BamC, BamD and BamE. BamA and BamD have been demonstrated to be essential in *E. coli* [10,12, 16].

Over a decade ago, another OM insertion machinery, the translocation and assembly module (TAM), was discovered [17]. The TAM consists of TamA, also an Omp85 family member that has structural similarity to BamA, and a second protein, TamB, which spans the periplasm and is anchored in the inner membrane (IM). TamA has three POTRA domains and interacts with TamB, which belongs to the AsmA family of proteins that were recently demonstrated to be involved in phospholipid transport in both *E. coli* [18] and *Pseudomonas aeruginosa* [19]. The TAM was first thought to function in the assembly of autotransporter proteins, specifically EhaA and Ag43 of *E. coli* and p1121 from *Citrobacter rodentium* [17]. However, the TAM only seems to be required for the assembly of a subset of autotransporters, including the inverse autotransporter intimin, whereas trimeric autotransporters are not substrates of the TAM [20, 21]. More research has since demonstrated the role of TAM in the assembly of fimbrial ushers [22,23] and TolC-like proteins, which form the OM channel of tripartite efflux pumps [24]. Although the BAM is self-sufficient in biogenesis of fimbrial ushers and autotransporters, the absence of TAM partially affects biogenesis of autotransporters and usher proteins [25]. A time-course assay was used to demonstrate efficient assembly of the usher protein FimD, completed in under 2 min in WT cells, while this took 240 min in the absence of TAM [23].

Since its initial discovery [17] – where its deletion in *C. rodentium* and *Salmonella enterica* was demonstrated to diminish virulence – TAM has been increasingly implicated in virulence in many other bacteria. TAM mutants of the fish pathogen *Edwardsiella tarda* have been shown to have reduced flagella formation with attenuated motility and invasion into host cells and even lowered host mortality [26,27]. Furthermore, *tamB* mutant strains of *Vibrio fischeri* showed a 3.7-fold fitness defect in a competition assay [26]. Again, *Brucella suis* mutants of MapB (the TamB orthologue) exhibit attenuated virulence in the mouse infection model and impaired macrophage infection capabilities [28]. Another TamB orthologue, MorC in *Aggregatibacter actinomycetemcomitans*, is necessary for maintaining proper membrane stability. Its C-terminal domain is required for maintaining membrane integrity and supporting normal responses to environmental stress [29]. Additionally, MorC is required for the correct secretion and assembly of fimbriae in *A. actinomycetemcomitans,* and its deletion results in reduced surface fimbriae and altered biofilm architecture [30].

Other virulence-associated phenotypes of the TAM include causing breaches in the permeability barrier of the OM under stress conditions [31], where the deletion of *tamA* led to increased sensitivity of carbapenem-resistant *Klebsiella pneumoniae* to vancomycin. The OM integrity impairment was further confirmed by a significantly higher 1-*N*-phenylnaphthylamine (NPN) fluorescence in the *tamA* mutant in a hypo-osmotic buffer [31]. It was recently demonstrated that the POTRA domains of TamA not only recruit TamB but also directly interact with OM lipids, particularly phosphatidylglycerol, to modulate local membrane properties while supporting efficient insertion of OMPs. This finding enhances mechanistic understanding of the TAM’s role in OM biogenesis and provides insight into how disruption of TAM components may impair bacterial virulence [32]. Among the seven AsmA-like proteins encoded by *P. aeruginosa*, four, namely YhdP, TamB, YdbH and PA4735, were shown to be redundantly essential for growth and OM barrier function [19]. The study demonstrated that these proteins contribute to glycerophospholipid transport to the OM, helping maintain the balance between phospholipids and LPSs that is critical for OM integrity [19]. However, apart from this AsmA-like protein analysis, which includes TamB as one of several paralogues examined [19], the role of the TAM in *P. aeruginosa* virulence has not been addressed.

*P. aeruginosa* is an opportunistic Gram-negative bacterium with myriad virulence factors that contribute to its success as a pathogen [33]. It is notorious for being resistant to antibiotics [34], thereby earning a spot in the World Health Organization’s bacterial priority pathogens list as a member of the high-priority group for research on and control of antimicrobial resistance [35]. In response to the pressing need for alternative therapeutics for the treatment of *P. aeruginosa* infection driven by the increasing emergence of antibiotic resistance [33]*,* and considering the essential role of the OM in *P. aeruginosa* along with the established involvement of TAM in OM biogenesis, we investigated the role TAM plays in virulence in *P. aeruginosa* and analysed its suitability as a drug target against this high priority pathogen.

## Methods

### Bacterial strains

The bacterial strains used in this study were *E. coli* DH5α and SM10π*pir*. The reference laboratory strain *P. aeruginosa* PA14 was used to generate all the knockout mutants constructed in this experiment. Bacteria were grown in lysogeny broth (LB)-Lennox at 37 °C unless otherwise stated. Room temperature (RT) is 20 °C. Antibiotics used were gentamicin (50 µg ml^−1^) for *P. aeruginosa* or (15 µg ml^−1^) for *E. coli* and tetracycline (100 µg ml^−1^) for *P. aeruginosa* unless indicated otherwise. Gene knockouts with subsequent replacement of genes with a tetracycline resistance cassette were achieved by allelic exchange via homologous recombination as described [36].

### Plasmid construction

Plasmid constructs used for the in-frame deletion of *tamA*, *tamB* and t*amAB* genes were obtained using the Platinum SuperFi II Polymerase (Thermo Fisher Scientific). Firstly, DNA fragments of ~1,600 bp upstream and downstream of the gene of interest were amplified from PA14 genomic DNA, including the start and stop codons of the gene of interest. Primers amplifying each fragment were designed to have an overhang complementary to the tetracycline-resistance cassette with flanking flippase recognition target sites (FRT-tet). FRT-tet was amplified from pFRT-tet 129 (Doug Mortlock Lab: Mortlock lab unpublished plasmids) and assembled via Gibson assembly [37] to obtain a sequence in the order upstream sequence-FRT-tet-downstream sequence in a pEXG2 vector [36] [3] to obtain the pEXG2 construct that was verified by colony PCR and whole-plasmid DNA sequencing by MicrobesNG (Birmingham, UK). Primers used for PCR and cloning are listed in Table S1 (available in the online Supplementary Material).

#### Construction of a *tam* knockout and introduction of FRT-tet as a selection marker

The pEXG2 construct was introduced into *P. aeruginosa* through mobilization using the strain SM10π*pir* by conjugation. Resultant transconjugants were selected on *Pseudomonas* isolation agar (PIA) infused with 50 µg ml^−1^ of gentamicin. Homologous recombination resulting in the subsequent curing of the plasmid backbone using sucrose-based selection as previously described [36] resulted in obtaining mutants with the gene of interest replaced with the FRT-tet cassette.

### Removal of tetracycline cassette from *tam* knockouts

To remove the Tet cassette, an arabinose-inducible plasmid encoding the *flp* recombinase was produced. This was done by introducing a temperature-sensitive *P. aeruginosa* origin of replication [38] into the plasmid pCMT-flp [39]. We also switched the selection marker from ampicillin to gentamicin. To increase the efficiency of the flippase expression in *P. aeruginosa*, we introduced the arabinose-inducible P_BAD_ promoter as well as the *araC* gene, amplified from the pBAD-HisA (Invitrogen), upstream of the *flp* gene to yield the plasmid pFLPGm-BAD. This was transformed into *P. aeruginosa* knockout mutants containing the FRT-Tet cassette by preparing chemically competent cells using the TSS method [40], transforming the plasmid using heat shock and recovering at 30 °C for 1 h before plating onto LB with 50 µg ml^−1^ gentamicin (Gm50) and glucose at 0.5% (w/v). After overnight growth at 30 °C, a transformant colony was inoculated in LB+Gm50+glucose 0.5%. Once the culture had reached mid-exponential phase, the medium was replaced with fresh LB+Gm50 containing 0.5% l-arabinose, after which the culture was incubated for 3 h to induce FLP expression. A 1 : 100 dilution of this culture was grown overnight on LB agar with arabinose at 0.05% but no gentamicin at 37 °C to cure the pFLPGm-BAD plasmid. A couple of colonies were inoculated in LB broth (no selection) to grow overnight at 37 °C. Dilutions were plated for individual colonies the following day. Colonies were then streaked out on Gm50, tetracycline 100 µg ml^−1^ or LB without antibiotic to look for clones sensitive to gentamicin and tetracycline. Loss of the Tet cassette and the *flp* plasmid was confirmed by PCR.

### Making complementation plasmids

The genes of interest were amplified from *P. aeruginosa* PA14 using primers binding ~100 bp up- and downstream of the genes of interest. The primers were designed such that they had overhangs complementary to pSRK-Gm [41] into which the fragment was assembled via Gibson assembly [37] and transformed into *E. coli* DH5α and plasmid DNA extracted using a plasmid miniprep kit (New England Biolabs) to obtain pSRK-Gm with a functional copy of *tamA* and *tamAB*. The construct was verified by Sanger sequencing (Source BioScience, Cambridge, UK). This was then transformed into electrocompetent cells of the corresponding knockout strains that had been cured of the Tet-resistance cassette. As a control strain, pSRK-Gm was transformed into electrocompetent cells of PA14-WT to obtain pSRK-PA14WT.

### Whole-cell and OM protein extraction

OM isolation was performed following the protocol of Leo *et al.* (2016) [42] with some adjustments. Briefly, bacteria were inoculated into 500 ml of LB and grown overnight at 37 °C with shaking at 180 r.p.m. Bacteria corresponding to 500 ml at an optical density at 600 nm (OD_600_) of 1.0 were pelleted by centrifugation for 10 min at 5,000 ***g***. The pellet was washed with 20 ml of 10 mM HEPES buffer at pH 7.4 and then resuspended in 10 ml of 10 mM HEPES, to which 0.1 mg ml^−1^ lysozyme, MgCl_2_ and MnCl_2_ at 10 mM and DNase I at 10 µg ml^−1^ were added. The cells were then lysed by sonicating on ice using a Soniprep 150 plus machine, 3×30 s with 1 min between pulses. The lysates were then centrifuged at 5,000 ***g*** for 10 min, followed by the supernatant being transferred to an SS-34 tube and centrifuged for 30 min at 25,000 ***g***. The supernatant was decanted, and the pellet was resuspended in 2 ml of 10 mM HEPES at pH 7.4 in addition to 2 ml of 2% (w/v) *N*-lauroyl sarcosine. The IMs were subsequently solubilized at RT with agitation for 30 min.

Following this, the tubes were centrifuged at 25,000 ***g*** for 30 min to pellet the OM, followed by a 10 min wash step with 5 ml 10 mM HEPES pH 7.4 at 25,000 ***g***. The pellet was then resuspended in 1 ml of 5% SDS with 5 mM EDTA in distilled water and stored at −80 °C.

### RNA extraction

Total RNA was extracted using Monarch total RNA Miniprep Kit (New England Biolabs T2010S) following the manufacturer’s extraction protocol for bacteria with some changes. Briefly, a subculture was made from overnight cultures and grown to mid-exponential phase (OD_600_ 0.5) at 37 °C and placed on ice immediately. Aliquots (2 ml) of cultures were put into pre-chilled 2-ml tubes to halt cellular processes. Samples were then centrifuged in a pre-chilled 4° C centrifuge at 12,000 ***g*** for 1 min. The pellets obtained were lysed enzymatically using 100 µl of 0.1 mg ml^−1^ lysozyme for 15 min, and EDTA was added to 10 mM. Following this step, the manufacturer’s extraction protocol was followed until RNA was eluted. Samples were then measured using the Nanodrop microspectrophotometer (Thermo Scientific NanoDrop 2000 Spectrophotometer), where samples were ensured to have an *A*_260_/*A*_280_ of 1.9–2.1 and an *A*_260_/*A*_230_ of 2.0–2.2. Samples were then stored at −80 °C and transported on dry ice to Novogene (Cambridge, UK) for RNA-sequencing (RNAseq).

### RNAseq and data analyses

Quality control, ribosomal RNA depletion, library preparation and sequencing (Illumina NovaSeq X) were done according to Novogene’s proprietary protocols, generating at least 6 Gbp sequence (paired end, 150 bp) data per sample.

The US web-based platform Galaxy (http://usegalaxy.org/) was used in initial analyses of transcriptomic data [43]. The genome sequence (fasta file) and annotations (gff file) for *P. aeruginosa* strain UCBPP-PA14 (acquired from https://ftp.ncbi.nlm.nih.gov/genomes/all/GCF/000/014/625/GCF_000014625.1_ASM1462v1/; 25 May 2025) were used. The quality of the sequence reads was assessed using FastQC Galaxy Version 0.74+galaxy1 [44]; no trimming of reads was needed as all sequence data were of high quality (Phred ≥30) and free of adapter sequences. Sequence reads were then mapped to the UCBPP-PA14 reference genome using HISAT2 (Galaxy Version 2.2.1+galaxy1) set to paired-end library, before counting the number of reads that mapped to genes using featureCounts (Galaxy v2.1.1+galaxy2). To test for differential gene expression between the Δ*tamA*, Δ*tamB* or Δ*tamAB* and WT samples, limma (Galaxy Version 3.58.1+galaxy0) [45] was run with the count data from featureCounts. Genes were considered significantly differentially expressed based on adjusted *P*-value<0.05 (Benjamini–Hochberg procedure) or *P*-value<0.05 and log_2_-fold change ≥1 (absolute value). Venny v2.1.0 [46] was used to create a Venn diagram of the significantly differentially expressed genes. DESeq2 (Galaxy Version 2.11.40.8+galaxy0) was used to generate normalized count data for the entire dataset.

Box plots and volcano plots were generated using the R package tidyverse v2.0.0. A Kyoto Encyclopedia of Genes and Genomes (KEGG)-based network analysis was undertaken using the significantly differentially expressed genes. To do this, KEGGREST v1.46.0 was used to download information on the KEGG entry for *P. aeruginosa* strain UCBPP-PA14. Nucleotide sequences of genes for the KEGG entry were downloaded and used to create a blastn database (blast v2.12.0+) against which the predicted genes of the National Center for Biotechnology Information (NCBI) -annotated genome used in the Galaxy-based work were searched. Hits were filtered based on the highest query coverage and identity, with this information used to map KEGG annotations to the NCBI annotations (KEGG – pau:PA14_31680=*tamA*, pau:PA14_31690=*tamB*; NCBI – PA14_RS12965=*tamA*, PA14_RS12970=*tamB*) (5,889 genes mapped in total). Data were analysed using SPIA v2.58.0 [47] to identify biological pathways activated or inhibited in the mutant strains compared with the WT.

### Mass spectrometry

Total protein (50 µg) was digested using trypsin following the modified S-Trap protocol (ProtiFi, USA) as described previously [48]. Dried peptides were reconstituted in 200 µl 5% acetonitrile/0.1% formic acid. Supernatants were transferred to high-recovery LC vials, and the autosampler was kept at 8 °C. Peptide separation was carried out on a Waters M-Class UPLC system equipped with a Phenomenex Kinetex XB-C18 column (2.6 µm, 15×0.3 mm) maintained at 30 °C, using a linear gradient of 3–35% acetonitrile (mobile phase B) over 12 min at a flow rate of 10 µl min^−1^, followed by washing at 80% B and re-equilibration to 3% B at 12 µl min^−1^. Three microlitres of each sample were injected in direct injection mode. Mass spectrometric analysis was conducted on a Sciex 7600 ZenoTOF operating in positive ion mode with data-independent acquisition (DIA) (zenoSWATH), employing a 25 ms TOF-MS scan and 65 variable SWATH windows (12 ms each) covering *m/z* 400–750, with a total cycle time of 1.146 s. Raw data files (.wiff, .wiff2, .wiff.scan) were processed using DIA-NN (version 1.9.2) [49] with a *P. aeruginosa* SwissProt FASTA database (downloaded Nov 2024), enabling library-free search and deep-learning-based spectra prediction. Label-free quantification was performed at a precursor FDR of 1.0%, with default DIA-NN parameters unless otherwise specified. Data were processed using AMICA v 3.01 [50]. Missing values were imputed using the minimum intensity method, and differential abundance was assessed using the limma statistical framework. Significantly changed proteins were considered at a threshold of log_2_-fold>2 and adjusted value *P*<0.05.

### Growth curves

Overnight cultures of all strains were set up in biological quadruplicate in LB (gentamicin was added at 50 µg ml^−1^ to complement strains). As a control, pSRK-PA14WT was used in comparisons with the complemented strains. The turbidity of the cultures was measured and adjusted to an OD_600_ of 0.1. An aliquot (2 µl) of diluted cultures was added to 200 µl of LB (with gentamicin at 50 µg ml^−1^ for complemented strains) in wells of a 96-well plate and sealed with a Breathe-Easy membrane (Sigma-Aldrich). LB (200 µl) was inoculated into separate wells as a sterility control. The plate was then incubated at 37 °C in a Cytation 3 plate reader with shaking, and OD_600_ was measured every 20 min for 24 h. Growth parameters were calculated using the Growthcurver package (version 4.3.3) [51] in R, which fits a logistic growth model to the complete OD₆₀₀ time series and extracts the maximal growth rate (µmax, h⁻¹) from the exponential phase of the fitted curve. OD₆₀₀ vs. time plots were generated in GraphPad Prism version 10.6.0 (796) (solely for visualization) and were not used for rate calculations.

### Growth competition of the *tam* mutant vs. knockouts

Mutant strains containing the Tet resistance cassette insert and PA14-WT were grown on PIA with 100 µg ml^−1^ of tetracycline and just PIA, respectively. Single colonies were inoculated into 5 ml of LB broth and grown overnight at 37 °C with 100 µg ml^−1^ of tetracycline for mutant strains. This was followed by measuring cultures and adjusting with LB to an OD_600_ of 1. Mutant strains and PA14 were inoculated into LB broth in a ratio of 1:1. Before the mixed culture was incubated, it was inoculated onto PIA-tet 100 and PIA only to ascertain that both strains were present at equal amounts and viable. The mixed cultures were retrieved the following day and serially diluted and plated for single colonies. Each culture was plated both on PIA only and on PIA-tet. Colonies were counted the following day, where the WT numbers were obtained by subtracting colonies on PIA-tet (mutant with Tet resistance cassette) from total colonies on PIA only (both mutants with Tet cassette and WT).

### Biofilm formation

Biofilm assay was performed as described by [52] with incorporated adjustments from the protocol of Merritt *et al.* [53].

Bacteria were inoculated into 5 ml of LB broth and grown overnight at 37 °C with shaking. Cultures were adjusted to an OD_600_ of 1 the following day and then diluted 1:100. Following this, 100 µl of the diluted cultures were aliquoted into a 96-well microtiter plate (Costar, Sigma-Aldrich) and incubated at 37 °C for 18 h. After this, the cultures were aspirated using a 1-ml pipette and washed three times with 200 µl of PBS to remove non-adherent cells. Excess PBS was removed by patting the plate firmly on a paper towel. The plate was then incubated for 1 h at 60 °C to fix adherent cells. Next, the biofilm was stained by adding 150 µl 1% (w/v) crystal violet followed by a 20-min incubation at RT. Plates were rinsed three times with distilled water and inverted to dry. Finally, 150 µl of 33% (v/v) glacial acetic acid was added to each well to solubilize the stained biofilm, after which the supernatants were transferred to a new plate and the OD_540_ was measured using a Cytation 3 plate reader.

### Swimming motility

Swimming motility assays were performed following the protocol of [54]. Five-times M8 solution was prepared with 64 g Na_2_HPO_4_·7H_2_O, 15 g KH_2_PO_4_ and 2.5 g NaCl in 1 l of water. An aliquot (200 ml) of 5×M8 solution was added to 800 ml of 0.3% autoclaved agar to yield a homogenous suspension. Glucose or casamino acid was added to a concentration of 0.2% and 0.5%, respectively. MgSO_4_ was then added to 1 mM and mixed thoroughly, and 25 ml of the mixture was poured into plates to solidify at RT. A sterile 10 µl pipette tip was dipped into an overnight culture of strains without the Tet resistance cassette (Tet-sensitive strains) and PA14-WT and stabbed into the agar layer of the plate carefully to avoid touching the base of the plate. Plates were incubated upright for 18 h, and the diameter of the cultures was then measured.

### Antibiotic susceptibility testing

Disk diffusion susceptibility testing was performed in accordance with EUCAST recommendations as described by Matuschek *et al.* [55]. Cartridges of antibiotic-containing discs were stored at −20 °C and allowed to equilibrate to room temperature prior to use. Overnight cultures of bacteria (quadruplicates for each strain) were obtained by inoculating a single colony into 5 ml of Mueller–Hinton broth and diluting to an OD_600_ of 0.1 the next day.

Mueller–Hinton agar plates of thickness 20 mm were inoculated with the diluted bacteria using a sterile cotton swab and discs were placed onto the inoculated media within 3 min of inoculation. Plates were incubated at 37 °C for 24 h, and the diameter (mm) of the zones of inhibition was measured.

### Chelator and detergent assay

Samples were prepared in the same way as for antibiotic susceptibility testing, as detailed above. Sterile empty 6 mm discs were soaked with 10 µl of 500 mM EDTA or 5 µl of 10% SDS and 10 µl of 500 mM EDTA.

### OM permeability testing

OM permeability testing was done using NPN as described previously [56]. Briefly, in a black microtiter plate, 50 µl of 40 µM NPN in 5 mM HEPES at pH 7.2 was aliquoted into wells and topped up with 50 µl of 20 mM EDTA. Bacterial suspension (100 µl) adjusted to an OD_600_ of 0.1 in 5 mM HEPES was added, and fluorescence was measured at 350 nm and 420 nm excitation and emission, respectively, within 3 min in a Cytation 3 plate reader. Control wells were prepared as follows: (a) 200 µl HEPES only, (b) HEPES (150 µl) plus NPN (50 µl), (c) bacterial suspension (100 µl) and HEPES (100 µl), and lastly (d) bacterial suspension (100 µl), NPN (50 µl) and HEPES (50 µl).

Results were expressed as relative fluorescence units, which were calculated as the fluorescence value of the cell suspension and NPN without the test substance subtracted from the corresponding value of the cell suspension with EDTA and NPN.

### *In vivo* testing of *tam* knockouts using *Galleria mellonella*

We used *G. mellonella* larvae (purchased from the *G. mellonella* Research Centre, University of Exeter, Exeter, UK) for infection assays. Mutant strains without the Tet resistance cassette insert or PA14-WT were cultured overnight at 37 °C, and subcultures were made the following day to grow until an OD_600_ of 0.4–0.5. Following this, cultures were diluted to specific c.f.u. ml^−1^ in PBS, based on a standard curve relating c.f.u. ml^−1^ to OD_600_. PBS was used as a negative control, while heat-killed PA14 (20-min incubation at 80 °C) served as a control for testing the toxicity of bacterial cell components. *Galleria* injections were done using 1 ml syringes with 27 G needles with a mechanical syringe pump (Cole-Parmer, St Neots, UK) at a rate of 13.21 ml h^−1^ and a volume of 10 µl per injection. Injections were made at the bottom left proleg into the hemocoel of the larvae. To verify the inoculum doses, 10 µl of the inoculum was diluted and plated. The health score of larvae was calculated following the scale of [57]. The health of larvae is directly proportional to the health score, whereby higher scores reflect healthy larvae.

## Results

### The *tam* mutants and their complemented strains

We first generated *tam* mutants in the *P. aeruginosa* PA14 background by standard marker exchange mutagenesis, where the *tamA* or *tamB* or the whole operon *tamAB* was replaced with a Tet resistance cassette flanked by FRT sites for later removal using the Flp recombinase [58, 59]. This approach enabled us to perform competition assays and select for mutants using tetracycline. The resultant mutants are referred to as ∆*tamA-tetR*, ∆*tamB-tetR* and ∆*tamAB-tetR*. Removal of the Tet resistance cassette with a novel Flp-encoding plasmid that replicates conditionally in *P. aeruginosa* resulted in mutants which were sensitive to tetracycline, namely ∆*tamA*, ∆*tamB* and ∆*tamAB* (Fig. S1A). Complemented strains with the genes *tamA* or *tamAB* cloned into the pSRKGm plasmid background [41] were dubbed ∆*tamA*::pSRK-*tamA* and ∆*tamAB*::pSRK-*tamAB,* respectively. We were unable to produce a plasmid to complement ∆*tamB*, despite several attempts. Unsuccessful attempts were also made by Ramezanifard *et al.* [60] to complement *tam* single mutants, *tamA* or *tamB*, in *Salmonella*. This could be due to the *tamA* stop codon overlapping with the start codon of *tamB* (Fig. S1B), leading to difficulties in expression or the construct being toxic. We, therefore, complemented ∆*tamB* with pSRK-*tamAB*.

### Growth rates of *tam* mutants and the WT are similar, but mutants are outcompeted in a mixed culture

**Fig. 1.**
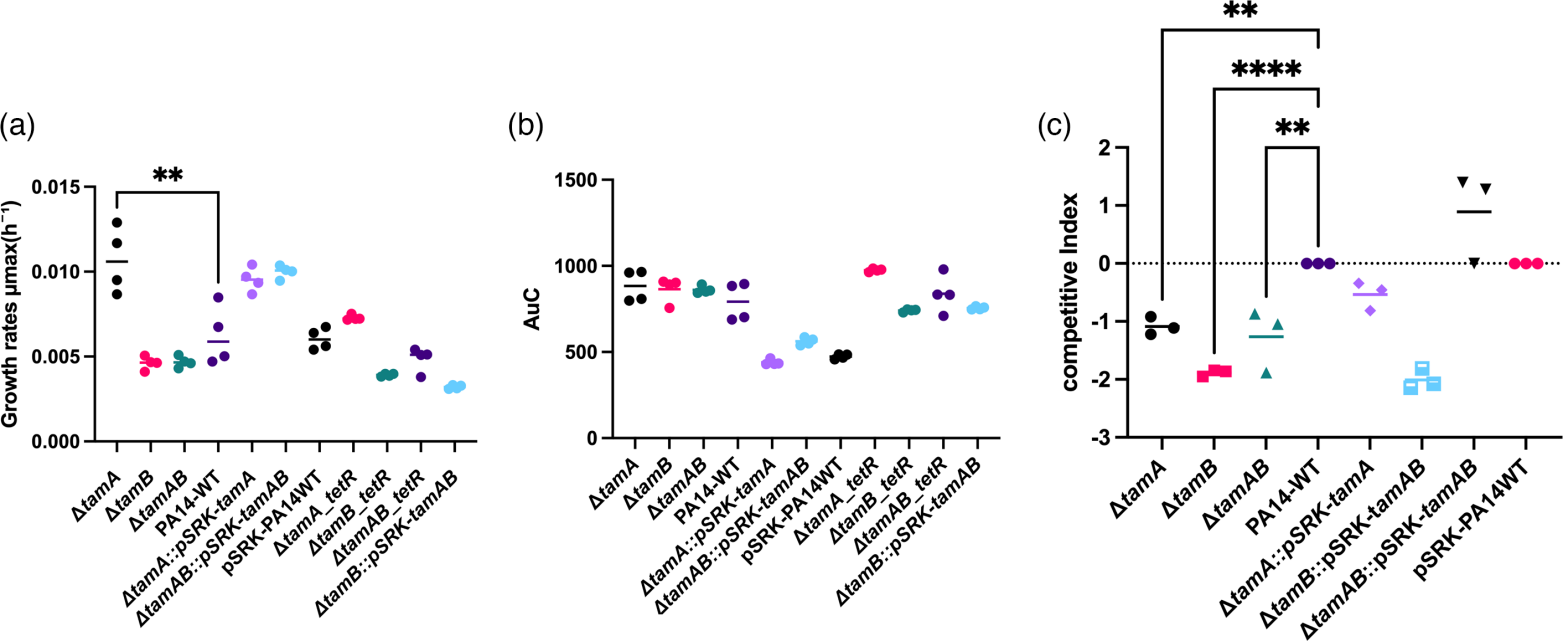
Growth and fitness of *tam* knockouts. (**a**) Growth rates of *tam* knockouts (∆*tamA,* ∆t*amB* and ∆*tamAB*), complemented strains (∆*tamA*::pSRK-*tamA*, ∆*tamB*::pSRK-*tamAB* and ∆*tamAB*::psRK-*tamAB*) and tetracycline-resistant strains (∆*tamA-tetR*, ∆*tamB-tetR,* and ∆*tamAB-tetR*). PA14-WT refers to the WT, and the pSRK-PA14WT refers to the WT with an empty pSRKGm plasmid. Growth rates were determined using the Growthcurver package in R, which fits a logistic growth model to OD_600_ values and derives the maximal growth rate during the exponential phase. (**b**) Area under the curve reflecting the total growth of strains. Growth was obtained using LB broth at 37 °C for 24 h (*n*=4), and growth rates and area under the curve were obtained using the Growthcurver package in R [51] from the growth curves in (Fig. S2). Statistical analysis was done for both panels (a) and (b) using one-way ANOVA with Dunnett’s post-hoc test; **P<0.005 compared to the WT (PA14-WT). The data shown represent one experiment using four biological replicates per strain. The experiment was repeated independently three times with similar results. (**c**) Growth competition between *tam* knockouts or complemented strains vs. WT[8] growth was performed in LB at 37 °C by growing equal amounts of each mutant and WT together. The bars indicate the competitive median. Competitive index was calculated as the ratio of c.f.u. of mutant to WT and plotted as log₁₀ (mutant/WT). Values below and above 0 indicate growth defect or growth advantage differential to the WT, respectively. Statistical analysis used was one-way ANOVA with Dunnett’s post-hoc test; **P<0.01, ****P<0.0001. Data shown as individual biological replicates, with horizontal lines indicating the median for each group.

We compared the growth rates of *tam* knockout strains and the WT by following growth over 24 h and generating growth curves (Fig. S2). Among all the strains, the ∆*tamA* mutant exhibited the fastest growth rate. The other strains tested all showed growth rates similar to the WT, in agreement with the lack of growth defect reported for *tamB* mutants [19]. To assess the relative fitness of the *tam* mutants compared to the WT (PA14-WT) under co-culture conditions, a competitive growth assay was performed by mixing an equal inoculum of the WT and mutant. This assay evaluates the ability of each strain to proliferate when competing for the same resources, providing a sensitive measure of subtle fitness differences that may not be apparent in monoculture. We hypothesized that if TAM contributes to OM integrity or protein assembly, the *tam* mutant would exhibit reduced fitness when co-cultured with the WT, resulting in a lower competitive index (CI<0). Consistent with this expectation, the *tam* mutants were significantly outcompeted by the WT (PA14-WT) (Fig. 1). Moreover, the CI was partially increased or fully restored when complemented with *tamA* or *tamAB*, respectively. However, complementation of the ∆*tamB* with pSRK-*tamAB* did not improve its CI in any way, possibly because of the presence of an additional copy of *tamA*.

**Deletion of *tamA* or *tamB* differentially impacts the OM proteome**

**Fig. 2.**
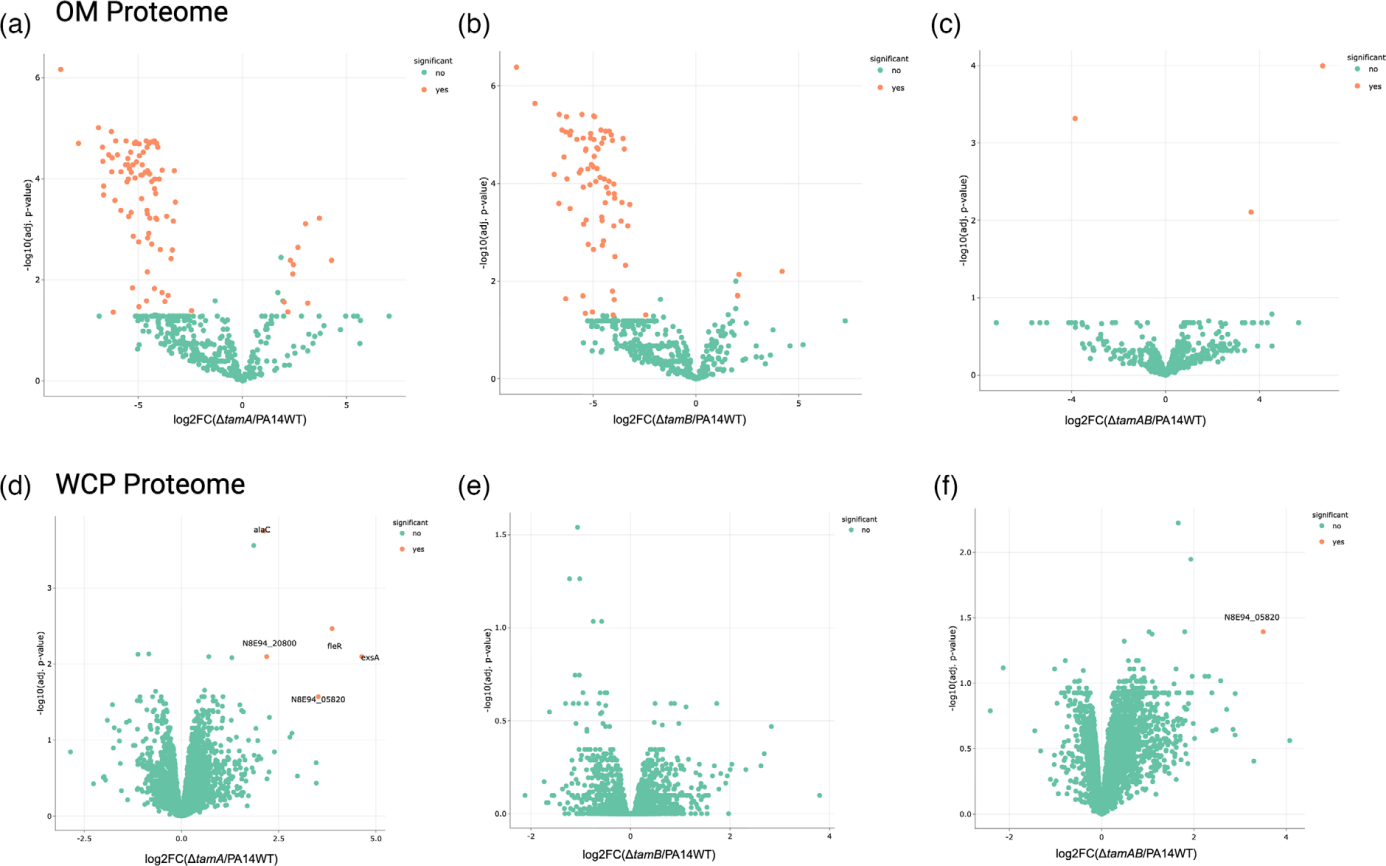
Differential envelope or whole cell protein abundances in whole cell proteins or outer membrane samples, respectively, of *tam* knockouts. Volcano plots showing the results of proteomic analysis of mutants and WT of (**a–c**) outer membrane protein extracts and (**d–f**) whole cell protein extracts. Orange dots represent proteins significantly upregulated or downregulated. Differentially abundant proteins identified by label-free quantification using DIA-NN and analysed with *amica* [50]. Proteins were filtered to retain those with at least two razor/unique peptides and a minimum of three MS/MS counts in at least one group. Missing values were imputed using the minimum intensity method, and differential abundance was assessed using the limma statistical framework. Significantly changed proteins were considered from log_2_-fold>2 and adjusted value *P*<0.05.

To verify whether deletions of *tamA*, *tamB* or *tamAB* affected the proteome of the mutants, we extracted the whole-cell protein (WCP) and the OMPs of the mutants and WT and analysed their proteome via mass spectrometry. In the OMP samples, we observed many significantly differentially expressed proteins between the single mutants compared to the WT (Fig. 2a, b). However, many of these were cytosolic proteins, which we assume are spurious contaminants in the OMP samples. We, therefore, manually filtered for cell-envelope proteins (Table S2), leaving out all the cytoplasmic proteins (Tables S3 and S4) from our analysis, especially because these cytoplasmic proteins were not significantly differentially expressed in the proteome analysis of the WCP (Fig. 2d, e). Proteomic profiling of the single mutants exhibited significant changes in proteins related to OM structure, transport and motility, with a few differences between each mutant (Table S2). In ∆*tamA*, differential expression was dominated by OM-associated proteins, including TonB-dependent receptors, TolC-like proteins, the OM assembly factor BamB and porins. Also, CheV and flagellar components found in their proteome suggest modulation of chemotaxis and motility pathways. There were many other flagellar components differentially expressed, which did not reach significant levels (Table S5); however, these changes possibly contribute to the negative impact of swimming motility in ∆*tamA* (see below).

In addition to the shared components like CheV, porins and TolC, *tamB* mutants showed an expanded set of differentially expressed proteins including CheW, a coupling protein that connects CheA to receptors to facilitate chemotaxis [61]; FlgG, which forms the distal rod as a drive shaft to transmit torque from the motor to the filament [62]; and PilY1, a type IV pilus biogenesis factor which mediates twitching motility [63]. Also, the ∆*tamB* proteome revealed a reduction of a putative secretion system protein and an RND efflux membrane fusion protein, suggesting a broader change in secretion and efflux systems.

There were surprisingly few differentially expressed proteins in the OMP proteome, however, when *tamAB* were deleted. The only three significantly different proteins were all cytoplasmic proteins, namely transketolase, DNA topoisomerase IV and DUF2599 domain-containing protein, all of which were not considered in our analysis as they were not significantly differentially expressed in the WCP.

In general, the WCP samples did not highlight many significant differences (Fig. 2d–f). It is worth noting that membrane proteins (including OMPs) are only a minor component of the WCP and are likely largely missed in these samples. For ∆*tamA* WCP, however, there were five proteins with increased expression – AlaC, N8E94_20800, N8E94_05820, FleR and ExsA – indicating an upregulation in components involved in metabolic pathways, transport systems, motility and secretion systems. ∆*tamAB* had only one significantly differentially upregulated protein, N8E94_05820, an uncharacterized RDD family membrane protein in *P. aeruginosa*. The RDD family constitutes a category of proteins characterized as a novel Na^+^(Li^+^,K^+^)/H^+^ antiporter in *Halobacillus andaensis* [64]. Na^+^(Li^+^,K^+^)/H^+^ antiporters maintain cellular homeostasis [65]. There were no significantly differentially expressed proteins in the WCP of ∆*tamB*. These findings highlight the minimal (∆*tamA*) to null significant (∆*tamB* or ∆*tamAB*) effect the *tam* has on the cytoplasmic protein profile of *P. aeruginosa* when deleted, as opposed to the impact it has on the outer membrane proteome when the single genes are deleted.

### Deletion of *tam* negatively impacts the biofilm formation abilities of mutants

Since there were no obvious differences in the proteome of the WCP of *tam* mutants, we decided to probe further by performing transcriptomic analyses to investigate differences, if any, at the mRNA level.

**Fig. 3.**
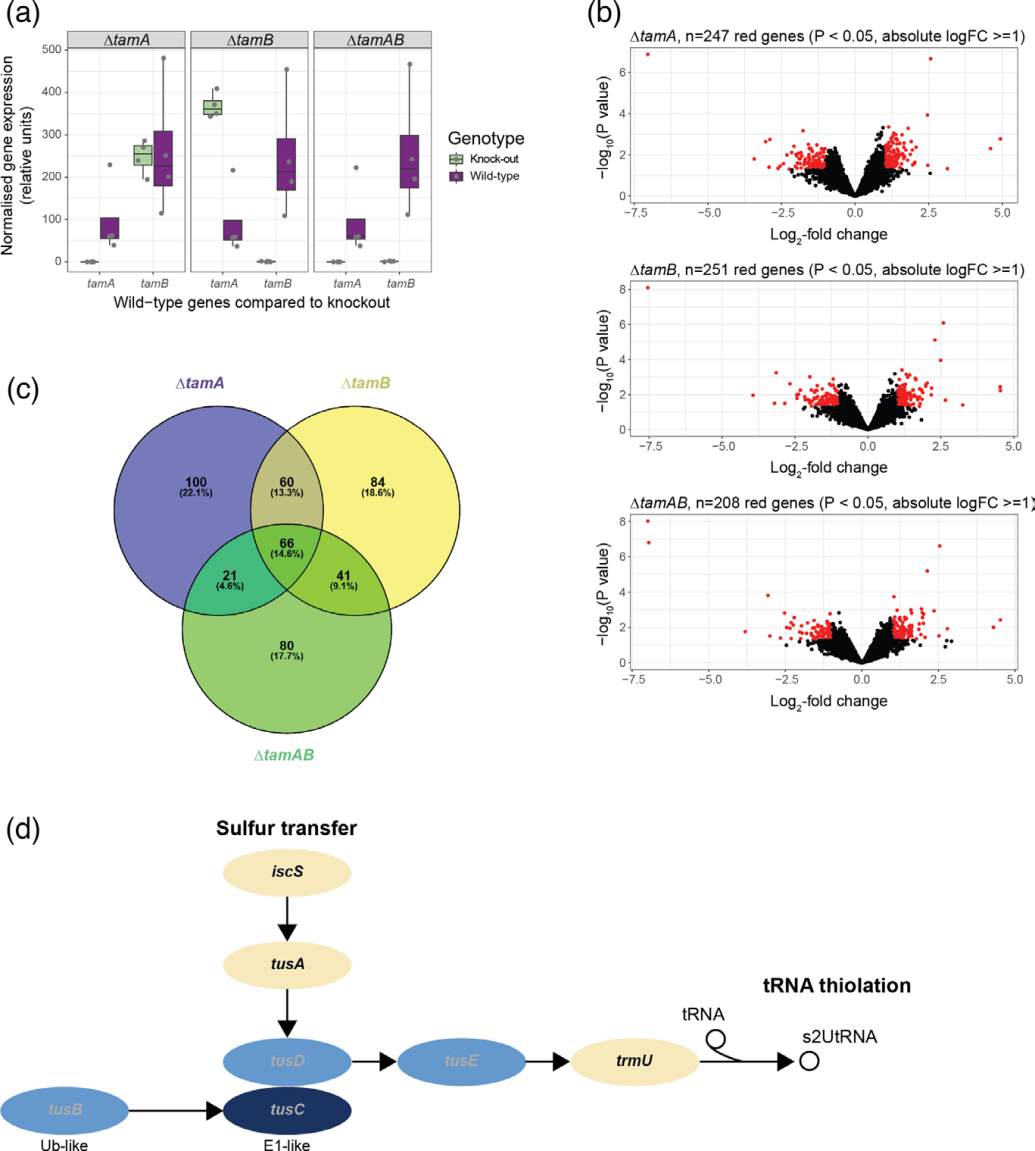
Analyses of transcriptomic data for the *ΔtamA*, *ΔtamB* and *ΔtamAB* mutants compared with the WT strain. (**a**) DESeq2-normalized data were plotted for the single- and double-mutant strains compared with the WT, confirming ablation of gene expression in the mutants. *n*=4 biological replicates per strain. (**b**) Volcano plots of log_2_-transformed normalized gene expression data. Significantly differentially expressed genes (*P*<0.05, absolute log_2_-fold change ≥1) are shown in red; all other genes are shown in black. (**c**) The significantly differentially expressed genes from (**b**) were compared and represented in a Venn diagram. (**d**) A KEGG-based Signalling Pathway Impact Analysis (SPIA) was carried out using genome data from *P. aeruginosa* strain UCBPP-PA14. The sulphur relay system was predicted to be significantly inhibited in the *ΔtamA* (data shown in image) and *ΔtamB* mutants. The darker the blue, the greater the negative log_2_-fold change (i.e. downregulation) in gene expression compared with the WT; yellow genes, non-significant.

Limma-based analyses of the transcriptomic data using an adjusted *P-*value threshold of 0.05 (Benjamini–Hochberg) showed that in the Δ*tamA* mutant, the expression of *tamA* was ablated (*P*=6.55×10^−4^; log_2_-fold change=−7.03927821) compared with the WT; in the *tamB* mutant, only the expression of *tamB* was ablated (*P*=4.77×10^−5^; log_2_-fold change=−7.54596815); and in the *tamAB* mutant, the expression of both *tamA* and *tamB* was ablated (*P*=4.72×10^−4^ and = 5.79×10^−5^, respectively; log_2_-fold changes −6.95671808 and −6.98747073, respectively) (Fig. 3a), demonstrating that the knockouts had been successful.

Using a less conservative approach to analyse the data (unadjusted *P-*value<0.05 and absolute log_2_-fold change ≥1), in Δ*tamA,* 247 genes (229 with KEGG annotations) were significantly differentially expressed; in Δ*tamB,* 251 genes (238 with KEGG annotations) were significantly differentially expressed; and in Δ*tamAB,* 208 genes (196 with KEGG annotations) were significantly differentially expressed compared with the WT (Fig. 3b). Sixty-six significantly differentially expressed genes were shared by the single- and double-knockout mutants (Fig. 3c).

Signalling Pathway Impact Analysis (SPIA) was used to map the significantly differentially expressed genes and their log_2_-fold change data onto KEGG pathways, to determine whether any of the pathways encoded by *P. aeruginosa* were potentially activated or inhibited by the gene mutations. At an adjusted *P-*value threshold of 0.05 (Benjamini–Hochberg), the sulphur relay system was significantly inhibited (*P*=0.047) in the Δ*tamA* mutant (Table S6). The expression of the *tusBCDE* genes associated with this pathway was significantly downregulated (Fig. 3d). Other pathways predicted to be inhibited in the Δ*tamA* mutant (but not significantly) included biofilm formation (in agreement with the phenotypic data below and linked to significant downregulation of genes associated with the type 6 secretion system and polysaccharide biosynthesis), quorum sensing, two-component systems and bacterial chemotaxis. The same pathways were also predicted to be inhibited in the Δ*tamB* and Δt*amAB* mutants, although only the sulphur relay system reached significance in Δ*tamB* at an adjusted *P-*value threshold of 0.1. SPIA, based on the 58 of 66 significantly differentially expressed genes with KEGG annotations that were shared by all 3 mutants, supported inhibition of all of these pathways except quorum sensing (Table S6).

### The OM integrity of mutants is impaired

**Fig. 4.**
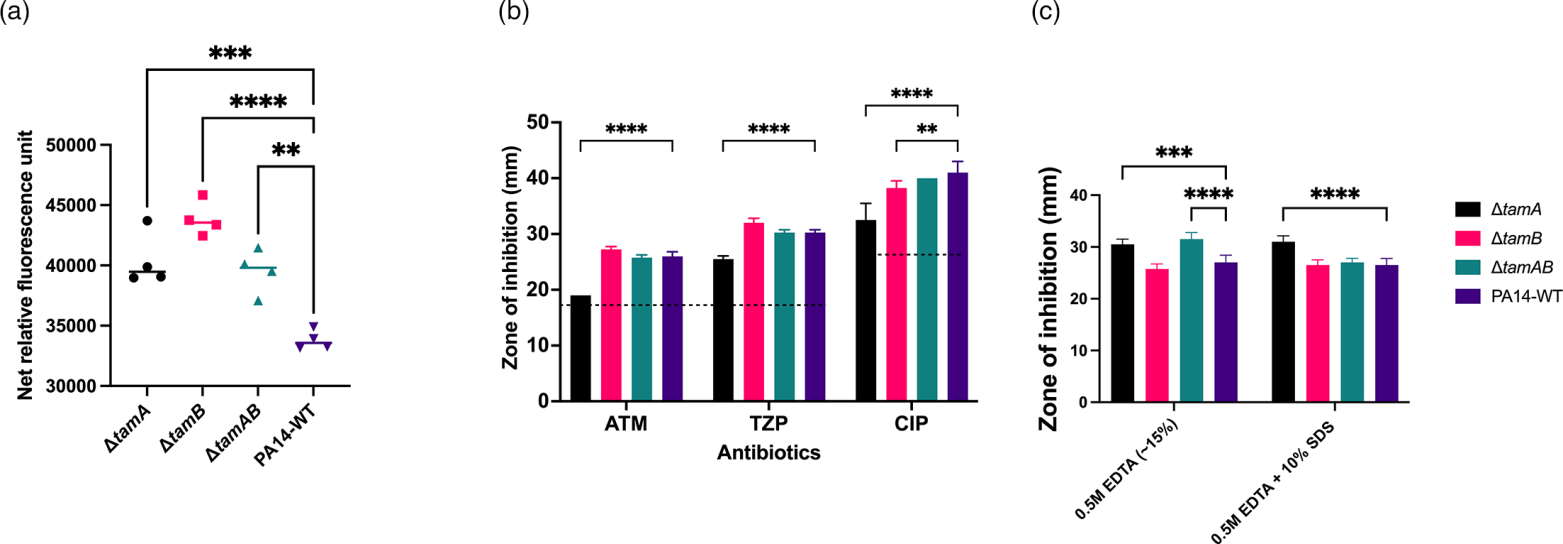
Assessing the OM integrity of *tam* mutants. (**a**) Oms of mutants are more permeable than the WT in the presence of EDTA. Permeability of the OM was assessed using an NPN assay [56]. Relative fluorescence units were calculated as the fluorescence value of the bacterial cell suspension and NPN without the test substance (no EDTA) subtracted from the corresponding value of the cell suspension with EDTA and NPN. **P<0.05; ***P<0.001; ****P<0.0001 determined by one-way ANOVA with Dunnett’s post-test. The data represent one experiment using four biological replicates per strain. The experiment was repeated independently three times with similar results. Horizontal lines indicate the median for each group. (**b**) Antibiotic susceptibility testing of *P. aeruginosa* PA14 WT and TAM knockout using disc diffusion assays. Antibiotics aztreonam (ATM), tazobactam–piperacillin (TZP) and ciprofloxacin (CIP) were used. Disk diffusion assay was performed and zones of inhibition were measured in mm. Black horizontal dashes across the graph represent EUCAST clinical breakpoints for resistance [66]. **P<0.01; ****P<0.0001. (**c**) Effect of chelators and detergents as measured by disc diffusion assays. The combination of EDTA and SDS enhances antimicrobial activity against ∆*tamA*. ***P<0.01; ****P<0.0001. Statistical analysis for both graphs B and C was determined by two-way ANOVA with Dunnett’s post-test, and the data shown represent one experiment using four biological replicates per strain. The experiment was repeated independently two times with similar results. Bars represent the mean with error bars indicating sd, where no error bar is visible, sd=0.

The integrity of the OM of mutants was assessed using NPN, which is a hydrophobic compound that fluoresces when it accesses the hydrophobic interior of the OM [56]. This only happens when the barrier function of the OM is impaired. Upon treatment of *tam* mutants or WT cells with EDTA followed by NPN, ∆*tamB* cells showed the highest fluorescence, followed by ∆*tamA* and ∆*tamAB*, all significantly higher than the fluorescence in the WT cells, showing the mutants’ OMs were more perturbed by EDTA and OM impairment among the *tam* mutants (Fig. 4a).

We also tested the effect of several antibiotics used to treat *P. aeruginosa* infections, as an impaired OM barrier could increase the sensitivity of the mutants. The antibiotics levofloxacin, tobramycin, meropenem, polymyxin B and colistin showed no increased efficacy in the mutants compared to the WT (Fig. S4); however, for aztreonam and tazobactam–piperacillin, the efficacy against the ∆*tamA* mutant was reduced, though still above the EUCAST clinical breakpoints [66]. Similarly, ciprofloxacin had significantly reduced efficacy against ∆*tamA* and ∆*tamB* (Fig. 4b). We also tested the effects of the chelator EDTA and the ionic detergent SDS and found that ∆*tamA* and ∆*tamAB* were significantly more susceptible to EDTA compared to the WT. The combination of EDTA and SDS enhanced antimicrobial activity against ∆*tamA*.

### Virulence markers in *tam* knockouts are attenuated

**Fig. 5.**
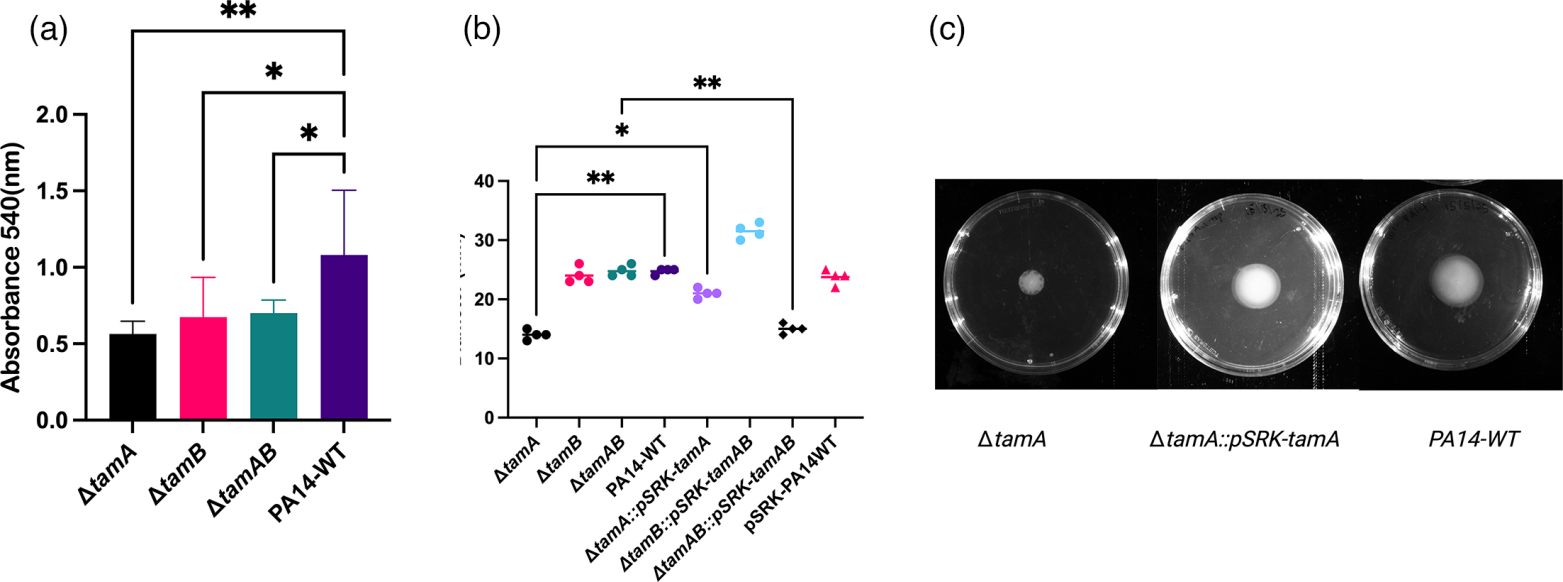
Impact of *tam* deletion on biofilm formation and swimming motility. (**a**) Biofilm formation of *P. aeruginosa* PA14 WT vs. *tam* knockouts. 96-well microtiter plates were inoculated with PA14-WT or *tam* mutants, and biofilm formation was quantified using the crystal violet assay. Absorbance was measured at 540 nm. Bars show the means of six biological replicates with error bars denoting standard deviations. *P*<0.05 and **P=0.001 (*P*<0.005). (**b**) Swimming motility in semi-solid agar, showing four biological replicates of each strain with lines depicting the median value. **P<0.005; *P<0.05. Statistical analysis obtained using one-way ANOVA with Dunnett’s post-test. Non-significant (ns) differences not displayed. (**c**) An image showing the swimming diameter of ∆*tamA* and its complemented strain ∆*tamA*::pSRK-*tamA* and WT. Images are representative of three independent experiments with the same results.

As *P. aeruginosa* is infamous for forming biofilm, we tested the mutants’ ability to form biofilm. Biofilm formation was reduced significantly in all mutants (Fig. 5a). Biofilm formation was partially restored in complemented strains as seen by an increase in biomass, although this did not reach statistical significance (Fig. S5A). Partial complementation of the phenotype is not surprising for complex, multifactorial phenotypes like biofilm formation, which depend on coordinated regulation of numerous structural and regulatory components [67,69]. Expression of *tam* from a plasmid may not fully reproduce native transcriptional control, localization or protein stoichiometry of the TAM complex, thereby limiting complete phenotypic recovery.

We proceeded to investigate whether swimming motility was attenuated, since we observed a reduction in the flagellar components in the mutants in the proteomic analysis. Swimming motility of ∆*tamA* was impaired significantly when assessed in a semi-solid agar motility assay, and this was significantly restored when complemented (Fig. 5b, c). The other mutants, ∆*tamB* and ∆*tamAB*, showed no reduction in their swimming abilities; however, when ∆*tamB* was complemented with *tamAB* in a pSRK background, this resulted in improved swimming motility recording the largest diameter (although not statistically significant) among all strains, suggesting that an extra copy of *tamA* might cause an additive improvement in ∆*tamB*::pSRK-*tamAB* (Fig. 5b). We also tested twitching motility, which is mediated by type 4 pili. However, no significant differences were observed between the *tam* mutants and WT (Fig. S5B).

### Reduced pathogenicity of *tamA* knockouts *in vivo*

**Fig. 6.**
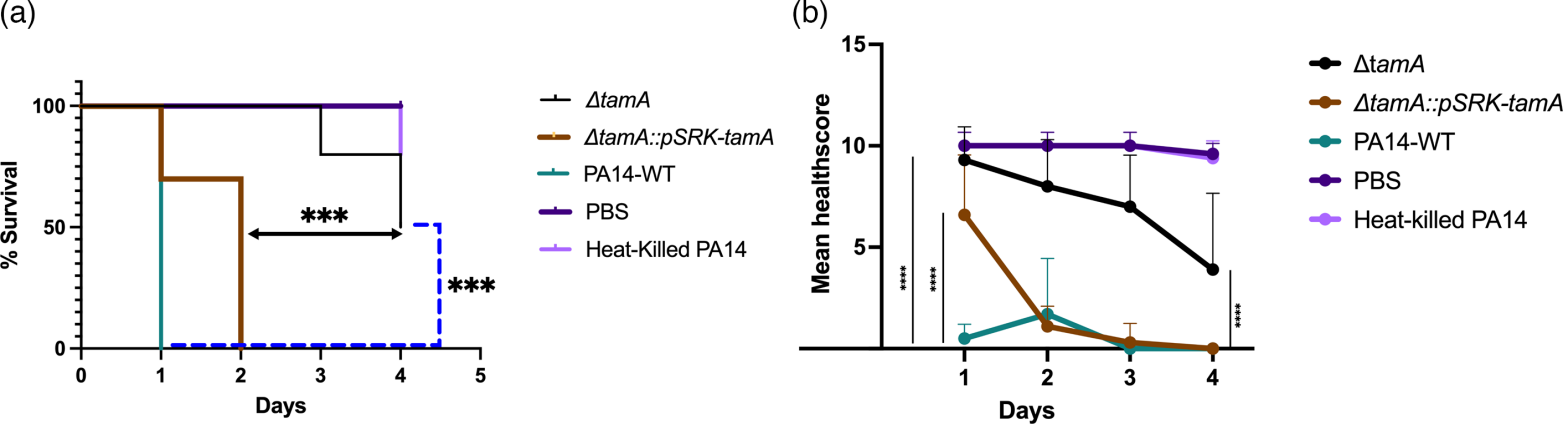
*tamA* knockout is less pathogenic. (**a**) Survival curve of *G. mellonella* post-infection. The differences in the survival curves of *∆tamA* and WT (PA14-WT) are significant, as shown by the blue dashes. The differences between ∆*tamA* and WT are indicated by the blue dashed line, whereas the difference between ∆*tamA* and ∆*tamA*::pSRK-*tamA* is indicated by the black double-headed arrow. Significance was determined via the Mantel–Cox test with a ***P<0.001. (**b**) Health score of *G. mellonella* post-infection. The health score of *n*=10 (where *n* represents technical replicates) was calculated daily for 4 days. Significant difference noted in ∆*tamA* strains from the first day through to the fourth day ****P<0.0001 (only first day and fourth day significance shown). For ∆*tamA*::pSRK-*tamA*, *a* significant difference observed on the first day (****P<0.0001) is displayed. From the second to fourth day, no significant difference is detected between ∆*tamA*::pSRK-*tamA and* WT (not displayed). Points with connecting lines show the mean health score of ten biological replicates, with error bars denoting standard deviations. Statistical analysis obtained by two-way ANOVA with Dunnett’s post-test.

To investigate whether the TAM plays a major role in *P. aeruginosa* virulence, we tested the virulence of the *tam* knockouts in a *G. mellonella* larval infection model [57]. Only Δ*tamA* mutants were seen to be significantly less pathogenic as infected larvae experienced reduced mortality (Fig. 6). Larvae infected with *ΔtamA*::pSRK-*tamA* experienced increased mortality, implying a restoration of the virulence potential in the complemented strain (Fig. 6a). The health score [57] of larvae infected with *tamA* mutants is significantly higher relative to the WT, while that of *ΔtamA*::pSRK-*tamA* is significantly higher on the first day but similar to the WT thereafter (Fig. 6b). Overall, the health score [57] of all the *tam* mutants was significantly higher after 24 h compared with the WT (Fig. S6C) even though the larvae died: the larvae were not as melanized when infected with the mutants, suggesting a slight reduction in virulence. The WT PA14 not only killed the *Galleria* within 24 h, but the tissue was also largely disintegrated (albeit disintegration was not considered in the health score), again suggesting that the WT bacteria were more virulent. Larvae infected with heat-killed bacteria resulted in some mortality on the fourth day, but this was not significant, nor was the health score significantly reduced. On the other hand, larvae injected with PBS did not result in any mortality or significant reduction in health score.

#### Discussion

The TAM consists of an integral membrane protein TamA connected by its three POTRA domains to an AsmA-like protein TamB, which spans the periplasm to the inner membrane. The TAM has been demonstrated to play important roles in the assembly of several *β*-barrel proteins into the OM [17,22,24, 70, 71].

Studies establishing the involvement of the TAM in the virulence of the opportunistic pathogen *P. aeruginosa* are lacking. Only one study has focused on *P. aeruginosa* as a model organism to investigate the involvement of the TAM in phospholipid transport [19]. Given that the outer membrane of *P. aeruginosa* contributes greatly to its virulence and overall success as a pathogen [72,73], compounded with the increasingly needed alternative drugs for treatment of *P. aeruginosa* infections due to antimicrobial resistance, we decided to investigate the TAM as a potential novel drug target.

We assessed the growth potential of *tam* mutants and WT and did not find significant differences between their growth profiles in LB or minimal media, except for ∆*tamA*, which had a slightly faster growth rate. Bacteria use a significant amount of their energy to synthesize flagella and drive motility [74]. Since there is reduced motility exhibited in ∆*tamA* strains, it is possible that energy that would have been used for motility is being directed towards faster growth.

We then decided to investigate the relative fitness of the knockout mutants in a competitive environment by co-culturing each mutant with the WT. We found the mutants were outcompeted by the WT. The inability of *tam* mutants to compete for nutrients equally in a co-culture growth environment with the WT could be due to the low level of porins and TonB-dependent transporters present both in ∆*tamA* and ∆*tamB* OM proteome (Table S2). Porins and TonB-dependent receptors facilitate the uptake of nutrients; their reduction in the mutants would result in inefficient competition for nutrients [75]. In a monoculture, however, the reduced transport capacity of mutants does not affect growth because of the lack of competition: the mutants are able to acquire sufficient nutrients for normal growth when the WT is not present, which can absorb limiting nutrients more efficiently.

The OM proteomes of the ∆*tamA* and ∆*tamB* are highly different from the WT. By contrast, the ∆*tamAB* mutant presented with a similar profile to WT, where differences observed were not significant (Figs 2a–c and S3). This could mean that single deletions or unbalanced expression of either protein TamA or TamB results in defects, possibly due to toxicities caused by the absence of one of the TAM subunits. Defects noted in the ∆*tamA* were relatively stronger compared to the ∆*tamB*, which could be due to TamB being one of the four functionally redundant (in terms of growth in *P. aeruginosa* and OM integrity) AsmA-like proteins, alongside YhdP, YdbH and PA4735 [19]. This aligns with similar findings in *E. coli* [18,76] where the absence of these AsmA-like proteins (excluding PA4735) results in phenotypes with impaired OM biogenesis [18,76] along with accumulated glycerophospholipids (GPLs) in the IM [76] and reduced flux of GPLs towards the OM [77].

Relatively minor effects, including lower growth capacity under competitive conditions, increased permeability to NPN in the presence of EDTA and reduced biofilm-forming abilities, observed from the deletion of *tamAB*, suggest that the TAM complex may not be essential for virulence in *P. aeruginosa*. Similar observations have been reported in *Salmonella*, where the loss of TAM has minimal impact on cell envelope integrity or infection phenotypes [60]. One possible explanation is that the BAM complex can compensate for the absence of TAM. BAM and TAM both contain Omp85-family components (BamA and TamA, respectively) with related *β*-barrel assembly functions [78,79], and biochemical reconstitution experiments have shown that TAM can catalyse OMP assembly in a manner comparable to BAM [25]. Also, genetic studies have demonstrated synthetic phenotypes when *tamAB* is deleted in *bam* mutant backgrounds, indicating functional overlap and partial redundancy between these systems [60]. In *Borrelia burgdorferi*, the TamB ortholog, BB0794, has been shown to interact with BamA, and mutants of BB0794 result in changes in cellular morphology and antibiotic sensitivity [80]. Also, it has recently been demonstrated that the TamB–BamA interaction in *B. burgdorferi* occurs between the TamB DUF490 domain and BamA POTRA2 and POTRA3 [81]. A similar connection between these systems has not been established in *Proteobacteria* but could be potentially plausible. The increased production of BamB and BamC in the OM proteome of single mutants could potentially be due to these lipoproteins assuming a greater role in OMP translocation and insertion in the absence of *tamA*. Deletion of *bamB* has been shown to not only affect changes to the OM permeability [82], but also to reduce pathogenicity and sensitize bacteria to antibiotics [83]. Similarly, the lack of BamC affects OM permeability and sensitizes bacteria to environmental stresses [84]. With the increase of these proteins in the absence of *tamA*, it could be inferred that the maintenance of the OM barrier is shifted towards BAM components.

Although there was mostly a reduction of structural components of flagella (Table S5) in the OM proteome of all mutants, we found that only the *tamA* mutant impacted swimming motility. Unlike our results, motility was significantly impaired in both *∆tamA* and *∆tamB* mutants of *E. tarda* [27]. The reduction of flagellar components would have been an explanation for attenuated biofilm formation ability among *tam* mutants, just as noted with the reduced surface fimbriae and resulting reduction in biofilm in *A. actinomycetemcomitans* MorC mutants [30]. However, the loss of flagellar proteins was not reflected among all the mutants.

KEGG-based SPIA of the transcriptomic data showed that biofilm formation pathways and quorum-sensing pathways were inhibited (non-significantly) in all mutants, reflecting the reduction in biofilm-forming abilities of the mutants. It should be noted that neither *tamA* (KEGG orthology K07278) nor *tamB* (KEGG orthology K09800) is directly implicated in the functioning of specific *P. aeruginosa* pathways. Instead, these genes are associated with the assembly of transporters and ion channels. As such, it is unsurprising that their ablation has relatively little effect on the transcriptome of *P. aeruginosa* PA14.

SPIA found only the sulphur relay system [85] to be significantly inhibited in the *ΔtamA* and *ΔtamB* mutants (Fig. 3d). TusA of this system has been implicated in biofilm formation in *P. aeruginosa* [86]. In *P. aeruginosa*, the sulphur-relay pathway includes the TusA-like persulphide carrier PA1006, which is vital for sulphur trafficking, molybdenum cofactor homeostasis, nitrate respiration and mature biofilm development [86,87]. Upon inhibition of TusBCDE, TusE is unable to stimulate sulphur transfer from TusA to TusD, essentially disrupting the sulphur relay system [85]. Disruptions to *tam* appear to be affecting tRNA thiolation via *tusBCDE*, potentially reducing translation, thereby impairing biofilm-forming abilities in the *tam* mutants. Work in eukaryotes shows that tRNA thiolation and translation are tightly coupled [88].

Channel proteins of the TolC family form the outer membrane component of tripartite efflux pumps. Structural analyses demonstrate that the efflux protein TolC in Gram-negative bacteria functions as a channel for antibiotic removal, influencing bacterial susceptibility and virulence [89]. Deletion of *tolC* in *E. coli* resulted in increased susceptibility to macrolides, tetracycline and quinolones [90]. TolC is downregulated sixfold in both single mutants, suggesting a potentially compromised TolC-mediated export of toxic compounds. We would, therefore, predict that these mutants will have reduced resistance to harmful compounds due to the impaired ability to expel toxic substances like antibiotics and detergents. Some significant differences were observed for detergents and chelators, suggesting that the OM integrity and the ability of the mutants to remove toxic compounds are lowered (Fig. 4c). However, the antibiotics tested against the mutants either showed a reduced effect or no difference compared with the WT (Figs 4b and S4). This is in contrast to the increased OM permeability demonstrated by the mutants in the NPN assay, suggesting that the TAM does play an important role in maintaining the barrier of the outer membrane, as its removal increases membrane permeability just as previously demonstrated in ∆*tamB* [18].

Consequently, if drugs are formulated to target the TAM, it should be noted not to use these drugs in combination with the antibiotics that elicit reduced susceptibilities in the TAM knockouts. Other drug/antibiotic combinations may need to be tested.

To ascertain what these phenotypes might mean *in vivo*, and to answer our question regarding the TAM’s suitability as a drug target, we infected *Galleria mellonella* larvae and found the ∆*tamA* mutant to be significantly less pathogenic as it had the best health outcome post-infection as well as reduced mortality compared to the WT (Fig. 6). This agrees with the findings of Jung *et al.* [31], who showed that Δ*tamA* mutants of *K. pneumoniae* were compromised in their ability to colonize the mouse intestine and easily removed by the host immune system. Similarly [17], Δ*tamA* or Δ*tamB* mutants of *C. rodentium* were defective in the colonization of mice. In the same study, it was shown that *S. enterica* SL1344 *tam* mutants became sensitive to human serum. Although larvae infected with Δ*tamB* died within 24 h in our study, they showed fewer signs of melanization and thus had a better health score, which suggests some level of attenuation of virulence. However, larvae infected with the *tamA* knockout displayed increased survival. This informed our preference for *tamA* as a candidate for drug development. As to what is potentially causing the reduced virulence, we hypothesize it could be due to reduced motility due to a reduction in flagella components. *P. aeruginosa* possesses a polar flagellum that functions primarily in motility. The flagellum has long been implicated in virulence in *P. aeruginosa* [91,92]. McManus *et al.* [93] demonstrated that motility is required for virulence in a rat model where non-motile isolates derived from the virulent, motile parental strain showed a marked loss of virulence. Similarly, Montie *et al.* [94] reported that the absence of flagella led to reduced virulence and resulted in improved survival outcomes in mice. More recent work by Garcia *et al.* [68] also supports the role of flagella in pathogenicity. In addition, flagellin produced from *fliC* has been shown to protect against *Pseudomonas* infection by enhancing humoral immune responses [95], making flagellin-based antigens promising candidates for vaccine development. Consistent with these observations, the reduction of flagellar components and the associated impairment in motility in *tamA* mutants has been shown to decrease the virulence potential of *P. aeruginosa*, as reflected by improved survival of *G. mellonella*.

It is important to recognize, however, that the relationship between flagella and virulence is context dependent. In specific host environments, particularly the cystic fibrosis lung, suppression or complete loss of flagella may provide a selective advantage by reducing immune detection and promoting long-term persistence [96,97]. Supporting this idea, recent evidence indicates that flagellum-deficient *P. aeruginosa* strains can display increased virulence in cystic fibrosis mouse models [98]. Thus, while flagella generally enhance virulence in acute infection settings, their absence may confer a fitness benefit in chronic or immune-adapted environments.

Altogether, our results point to the importance of the *tamA* or *tamB* in OMP biogenesis in *P. aeruginosa*. However, neither GPL nor lipid composition analysis was performed, and, therefore, potential effects on GPL transport or biosynthesis were not assessed, though a previous study indicated a role for the TAM in OM GPL transport in *P. aeruginosa* [19]. In addition, the impaired motility phenotype observed in the *tamA* mutant is consistent with a reduction in the abundance of flagellar-associated proteins. Also, TAM mutants have a reduced biofilm-forming ability and perform poorly in a competition environment. These findings together lead us to propose that ∆*tamA* is a potential drug target against *P. aeruginosa*. However, further investigation is required to understand why, despite there being an increase in OM permeability in the TAM mutants, their susceptibility to antibiotics is unchanged or lower.

The OM of Gram-negative bacteria poses a major hurdle against the discovery of new antibiotics aimed at treating infections caused by multidrug-resistant bacteria. OM-associated proteins are, therefore, attractive novel targets for drugs. With advancements in the novel drugs targeting BamA, for example, darobactin [99] and monoclonal antibodies [100], similar approaches could be used towards novel drugs against TamA.

## Supplementary material

10.1099/mic.0.001674 Supplementary Material

## Abbreviations

BAM: *β*-barrel assembly machinery
DIA: data-independent acquisition
FRT-tet: tetracycline resistance cassette with flanding flippase recognition targets sites
GPL: glycerophospholipid
IM: inner membrane
KEGG: Kyoto Encyclopedia of Genes and Genomes
LB: lysogeny broth
LPS: lipopolysaccharide
NPN: 1-*N*-phenylnaphthylamine
OM: outer membrane
OMP: outer membrane protein
PIA: *Pseudomonas* isolation agar
POTRA: polypeptide transport-associated motif
RT: room temperature
SPIA: Signalling Pathway Impact Analysis
TAM: translocation and assembly module
WCP: whole-cell protein
