# Supplementary material for: The Omp85 family protein, TamA, exhibits characteristics of a suitable drug target against Pseudomonas aeruginosa
Source: Microbiology (Reading). 2026 Apr 21;172(4):001674. doi: 10.1099/mic.0.001674 (PMC13098762; doi:10.1099/mic.0.001674)
Supplement: Supplementary Material [file mic-172-01674-s001.pdf]

SUPPLEMENTARY MATERIALS

Supplementary Table 1: List of primers used in this study

Primer	Sequence	Comments
TamAUP_FR	CTCGAGCCCGGGGATCCTAAATGACCATGCAACCCTTCGT	For amplifying fragment upstream of *tamA*
TamAUP_Rev	GCCGAATTCCAGCACACTCATTCAGGTCGCCGCCGG	For amplifying fragment upstream of *tamA*
TamADS_FR	AGCCGAATTCTGCAGATTGAGTGAAGGCGCTGAAGATC	For amplifying fragment downstream of *tamA*
TamADS_Rev	CTGCAGGTCGACTCTAGAGGAATAGCGCCGGCTGG	For amplifying fragment downstream of *tamA*
TamAcomp_FR	CACAGGAAACAGCATATGATCACCAGTTGCCGATGCTC	For amplifying tamA locus for insertion into pSRK-Gm
TamAcomp_Rev	TTCTAGAGCGGCCGCCAAGTTGCGTACCGTCCTGCG	For amplifying tamA locus for insertion into pSRK-Gm
TamBUP_FR	CTCGAGCCCGGGGATCCAGGCCGACGGCGCGCG	For amplifying fragment upstream of *tamB*
TamBUP_Rev	GCCGAATTCCAGCACACTCACTCACAGTTCTGGCCCC	For amplifying fragment upstream of *tamB*
TamBDS_FR	AAGCCGAATTCTGCAGATTGACGGATGCGCTGATACGGG	For amplifying fragment downstream of *tamB*
TamBDS_Rev	CTTCTGCAGGTCGACTCTAGAGGCGATCCGCCCGGAGT	For amplifying fragment downstream of *tamB*
pEXG2_FR	TCTAGAGTCGACCTGCAGAAG	For amplifying pEXG2 backbone
pEXG2_Rev	GGATCCCCGGGCTCGAG	For amplifying pEXG2 backbone
FRT-TET_FR	TGTGCTGGAATTCGGCTTGAAGTTC	For amplifying FRT-TET from pFRT-Tet 129
FRT-TET_Rev	ATCTGCAGAATTCGGCTTGAAGTT	For amplifying FRT-TET from pFRT-Tet 129
pSRK_FR	TGGCGGCCGCTCTAGAAC	For amplifying pSRK-Gm back bone
pSRK_Rev	ATGCTGTTTCCTGTGTGAAATT	For amplifying pSRK-Gm back bone
pCMTf	AAC CAG GCG TTT AAG GGC ACC AA	For inserting the mSF-ts1 ori into pCMT
pCMTr	GCT ACG CCT GAA TAA GTG ATA ATA AGC	For inserting the mSF-ts1 ori into pCMT
mSF-pCMTf	CAC TTA TTC AGG CGT AGC CTG CAG AAA GGC AGG CCG GG	for amplifying the mSF-ts1 promoter for insertion into pCMT
mSF-pCMTr	G CCC TTA AAC GCC TGG TT CCT CTC AGG CGC CGC TG	
pCMT-GmCassF	ACGCGATGGATATGTTCT CAG AGC GCT TTT GAA GCT GAT	for amplifying the Gm cassette for insertion into pCMT
pCMT-GmCassR	CAGCCCCATACGATATAA GAT CCC CTG ATT CCC TTT GTC	
pCMTf3	TTATATCGTATGGGGCTGACTTC	for amplifying pCMT to switch the antibiotic cassettes
pCMTr3	AGA ACA TAT CCA TCG CGT CCG	
Gm-araBADf	GAA TCA GGG GAT CTT A GAG CTG CAT GTG TCA GAG G	for amplifying the P_{BAD}-*araC* insert
CMT-araBADr	GAT GGT GGT GAT GAT GCA T GGT TAA TTC CTC CTG TTA GTA	
GmF	TAAGATCCCCTGATTCCCTTTG	for amplifying the vector to insert P_{BAD}-*araC*
pCMTr	ATG CAT CAT CAC CAC CAT CAC	

Supplementary Table 2. Outer membrane proteins (OMP) expressed at significant levels in Δ*tamA* and Δ*tamB* mutant proteome compared with WT.

	**UNIPROT PROTEIN ID**	**PROTEIN DESCRIPTION**	**logFC**
ΔtamA			
	A0A7M2ZZQ8_PSEAI	Channel protein TolC	-6.287048852
	A0A0H2Z834_PSEAB	Flagellar hook-associated protein 2	-5.475610636
	A0A0H2ZDT9_PSEAB	Chemotaxis protein CheV	-5.362046911
	A6V673_PSEA7	Porin	-5.122999336
	A0A0H2ZH83_PSEAB	TolC family protein	-5.117572791
	A0A0H2ZGG4_PSEAB	Putative lipoprotein	-4.953177921
	A0A0H2Z6U8_PSEAB	TonB-dependent receptor	-4.86532359
	A0A0H2ZHF8_PSEAB	Putative outer membrane protein	-2.439195285
	A0A0H2ZJN1_PSEAB	Outer membrane assembly lipoprotein YfiO	2.421652005
	YFIB_PSEAE	Outer-membrane lipoprotein YfiB	3.03583481
	Q9HXJ7	Outer membrane protein assembly factor BamB	4.282713113
ΔtamB			
	A0A2R4B1G8_PSEAI	Putative porin	-7.824455707
	A0A643IRS3_PSEAI	Porin	-6.642985962
	A0A0H2ZA96_PSEAB	Transporter	-6.511634235
	A0A0H2Z917_PSEAB	Putative purine-binding chemotaxis protein CheW	-6.326043707
	A0A7M2ZZQ8_PSEAI	Channel protein TolC	-6.287048852
	A0A9Q9N8P1_PSEAI	Putative secretion system protein	-6.123551896
	A0A0H2Z834_PSEAB	Flagellar hook-associated protein 2	-5.475610636
	A0A0H2ZDT9_PSEAB	Chemotaxis protein CheV	-5.362046911
	A6V673_PSEA7	Porin	-5.122999336
	A0A072ZBY7_PSEAI	TolC family protein	-5.117572791
	A0A0H2ZCQ2_PSEAB	Putative TonB-dependent receptor	-4.967871431
	A0A0H2ZFL2_PSEAB	Putative outer membrane protein	-4.209489762
	PILY1_PSEAB	Type IV pilus biogenesis factor PilY1	-4.058229298
	A0A0H2Z825_PSEAB	Flagellar basal-body rod protein FlgG	-3.94962816
	A0A0H2ZGA2_PSEAB	RND efflux membrane fusion protein	-3.313169301
	BAMB_PSEAE	Outer membrane protein assembly factor BamB	1.941419858
	A6V2I3_PSEA7	Lipoprotein, putative	2.027421614

Supplementary table 3. List of all differentially expressed proteins (unfiltered)in *ΔtamA* proteome which are significant

Uniprot Id	Protein Description	LogFC ΔtamA vs PA14
A0A077JXD3_PSEAI	Fucose-binding lectin	-7.865929338
A0A0H2ZJM2_PSEAB	alcohol dehydrogenase	-6.907702836
A0A0H2ZH89_PSEAB	Uncharacterized protein	-6.711833929
PHZF_PSEAE	Trans-2,3-dihydro-3-hydroxyanthranilate isomerase	-6.69507018
ACNA_PSEAE	Aconitate hydratase A	-6.661768621
A0A0H2ZE94_PSEAB	Alkyl hydroperoxide reductase	-6.657786159
PNP_PSEAB	Polyribonucleotide nucleotidyltransferase	-6.413638591
CATA_PSEAE	Catalase A	-6.410061983
A0A7M2ZZQ8_PSEAI	Channel protein TolC	-6.287048852
A0A0H2Z9D0_PSEAB	oxoglutarate dehydrogenase (succinyl-transferring)	-6.268308894
A0A0H2ZG18_PSEAB	Putative nonribosomal peptide synthetase	-6.250094362
A0A509JM65_PSEAI	NAD-glutamate dehydrogenase	-6.198699009
ODO2_PSEAE	Dihydrolipoyllysine-residue succinyltransferase component of 2-oxoglutarate dehydrogenase complex	-6.117983608
ILVC_PSEAB	Ketol-acid reductoisomerase (NADP(+))	-6.079632011
A0A077SYW5_PSEAI	Cyanide-insensitive cytochrome bd quinol oxidase subunit I	-5.996137321
SYV_PSEAE	Valine--tRNA ligase	-5.834216731
PURA_PSEAB	Adenylosuccinate synthetase	-5.826351974
A0A0H2Z6K8_PSEAB	2,3-dehydroadipyl-CoA hydratase	-5.606538874
RISB_PSEAB	6,7-dimethyl-8-ribityllumazine synthase	-5.583834168
RL10_PSEAB	Large ribosomal subunit protein uL10	-5.53846464
A0A0H2ZFE1_PSEAB	Universal stress protein	-5.505684751
ALGR_PSEAE	Positive alginate biosynthesis regulatory protein	-5.495513944
A0A0H2Z834_PSEAB	Flagellar hook-associated protein 2	-5.475610636
ARCA_PSEAE	Arginine deiminase	-5.458409552
RL31_PSEAB	Large ribosomal subunit protein bL31	-5.422771312
A0A0H2ZDT9_PSEAB	Chemotaxis protein CheV	-5.362046911
A0A0H2ZDU5_PSEAB	Adenylate cyclase	-5.337051737
EFP_PSEAB	Elongation factor P	-5.332813598
RL16_PSEAB	Large ribosomal subunit protein uL16	-5.273408281
SYP_PSEAB	Proline--tRNA ligase	-5.25084362
A0A0H2ZCR1_PSEAB	R body protein RebB-like protein	-5.23645196
A0A072ZNQ8_PSEAI	Glycine zipper 2TM domain-containing protein	-5.179865506
RL32_PSEAB	Large ribosomal subunit protein bL32	-5.148210906
	Porin	-5.122999336
A0A072ZBY7_PSEAI	TolC family protein	-5.117572791
MQO1_PSEAE	Probable malate:quinone oxidoreductase 1	-5.112669247
SUCD_PSEAE	Succinate--CoA ligase [ADP-forming] subunit alpha	-5.080746053
EFTS_PSEAB	Elongation factor Ts	-4.978803416
CH60_PSEAB	Chaperonin GroEL	-4.965385073
A0A0H2ZGG4_PSEAB	Putative lipoprotein	-4.953177921
A0A071KW46_PSEAI	LemA family protein	-4.943843523
A0A0F7QY13_PSEAI	TonB-dependent receptor	-4.86532359
MURA_PSEAB	UDP-N-acetylglucosamine 1-carboxyvinyltransferase	-4.836392269
FTNA_PSEAE	Bacterial ferritin	-4.814124726
A0A0H2ZBY5_PSEAB	DUF883 domain-containing protein	-4.80517573

AROC_PSEAB	Chorismate synthase	-4.755118918
A0A0H2Z9V8_PSEAB	Putative universal stress protein	-4.665669955
RS3_PSEAB	Small ribosomal subunit protein uS3	-4.659004462
A0A367MEB1_PSEAI	Ferrichrome-iron receptor	-4.614810069
A0A0H2ZI23_PSEAB	Transcription termination/antitermination protein NusA	-4.602545961
SECY_PSEAE	Protein translocase subunit SecY	-4.577720346
A0A0H2Z8M2_PSEAB	Small-conductance mechanosensitive channel	-4.57341222
RRAAH_PSEAB	Putative 4-hydroxy-4-methyl-2-oxoglutarate aldolase	-4.562684972
VFR_PSEAE	cAMP-activated global transcriptional regulator Vfr	-4.559808048
LAP_PSEAB	Aminopeptidase	-4.547764704
A0A0H2ZC12_PSEAB	Autotransporter domain-containing protein	-4.545814389
PYRG_PSEAB	CTP synthase	-4.492035666
A0A0H2Z999_PSEAB	Putative outer membrane receptor protein	-4.482399522
A0A6A9KBX7_PSEAI	TonB-dependent siderophore receptor	-4.465705573
OTCC_PSEAE	Ornithine carbamoyltransferase, catabolic	-4.45061828
IDH_PSEAB	Isocitrate dehydrogenase [NADP]	-4.443132552
A0A0H2Z9D1_PSEAB	Transcriptional regulator CysB	-4.383737076
RL28_PSEAB	Large ribosomal subunit protein bL28	-4.35198133
A0A0H2ZDI1_PSEAB	Putative zinc-binding dehydrogenase	-4.350925315
A0A0H2ZK67_PSEAB	ATP-dependent RNA helicase RhlE	-4.225342229
A0A0H2ZLD6_PSEAB	Nitrite reductase	-4.223383052
PHOP_PSEAE	Two-component response regulator PhoP	-4.215820831
A0A0H2ZFL2_PSEAB	Putative outer membrane protein	-4.209489762
A0A0H2ZI19_PSEAB	DUF748 domain-containing protein	-4.204256511
A0A0H2ZKH1_PSEAB	Efflux RND transporter periplasmic adaptor subunit	-4.161469157
FLEQ_PSEAE	Transcriptional regulator FleQ	-4.152447594
A0A0H2Z762_PSEAB	Exonuclease VII large subunit C-terminal domain-containing protein	-4.115454865
NUOH_PSEAB	NADH-quinone oxidoreductase subunit H	-4.105216058
PILY1_PSEAB	Type IV pilus biogenesis factor PilY1	-4.058229298
SSB_PSEAE	Single-stranded DNA-binding protein	-3.989148826
HEM2_PSEAE	Delta-aminolevulinic acid dehydratase	-3.938687862
RS14_PSEAB	Small ribosomal subunit protein uS14	-3.853930137
A0A0H2ZKT8_PSEAB	Transketolase	-3.849767603
A0A0H2ZL81_PSEAB	Iron-sulfur cluster carrier protein	-3.717451582
A0A0H2ZKE8_PSEAB	C-type cytochrome	-3.632774412
RL30_PSEAB	Large ribosomal subunit protein uL30	-3.565629276
A0A0H2ZIJ0_PSEAB	Carbamoyltransferase	-3.421879316
RS20_PSEAB	Small ribosomal subunit protein bS20	-3.360146968
A0A0H2ZGA2_PSEAB	RND efflux membrane fusion protein	-3.313169301
HCP1_PSEAE	Protein hcp1	-3.264978678
FTSA_PSEAE	Cell division protein FtsA	-3.215474622
A0A0H2ZHF8_PSEAB	Putative outer membrane protein	-2.439195285
A0A0H2Z8K3_PSEAB	Sn-glycerol-3-phosphate transporter	-1.306013263
A0A6A9JVT0_PSEAI	DUF533 domain-containing protein	1.705979976
A0A0H2ZDL5_PSEAB	Peptidyl-prolyl cis-trans isomerase	1.857560154
A0A0H2ZES8_PSEAB	Putative lipoprotein	1.945347733
A0A0H2ZHP1_PSEAB	Paraquat-inducible protein B-like protein	2.004496465
ATPA_PSEAB	ATP synthase subunit alpha	2.187650967
A0A3M5EB07_PSEAI	BON domain-containing protein	2.308352261
A0A0H2ZJN1_PSEAB	Outer membrane assembly lipoprotein YfiO	2.421652005
A0A0H2Z890_PSEAB	Putative lipoprotein	2.451576231
A0A0H2ZDH6_PSEAB	General secretion pathway protein D	2.669518389
YFIB_PSEAE	Outer-membrane lipoprotein YfiB	3.03583481
A0A0H2ZIC6_PSEAB	OsmE family transcriptional regulator	3.138522732

A0A069QEN7_PSEAI	Chromosome partitioning protein ParA	3.700437505
BAMB_PSEAE	Outer membrane protein assembly factor BamB	4.282713113

Supplementary table 4. List of all differentially expressed proteins (unfiltered)in $\Delta tamB$ proteome which are significant

UniProt Id	Protein Description	LogFC $\Delta tamB$ vs PA14
A0A0H2ZG05_PSEAB	Putative porin	-7.824455707
ALGP_PSEAE	Transcriptional regulatory protein AlgP	-6.889544021
ACNA_PSEAE	Aconitate hydratase A	-6.661768621
A0A643IRS3_PSEAI	Porin	-6.642985962
A0A0H2ZA96_PSEAB	Transporter	-6.511634235
PNP_PSEAB	Polyribonucleotide nucleotidyltransferase	-6.413638591
CATA_PSEAE	Catalase A	-6.410061983
A0A0H2ZE85_PSEAB	Fucose-binding lectin	-6.33127282
A0A0H2Z917_PSEAB 9	Putative purine-binding chemotaxis protein CheW	-6.326043707
A0A7M2ZZQ8_PSEAI	Channel protein TolC	-6.287048852
A0A0H2Z9D0_PSEAB	oxoglutarate dehydrogenase (succinyl-transferring)	-6.268308894
A0A0H2ZC71_PSEAB	Putative secretion system protein	-6.123551896
ODO2_PSEAE	Dihydrolipoyllysine-residue succinyltransferase component of 2-oxoglutarate dehydrogenase complex	-6.117983608
ILVC_PSEAB	Ketol-acid reductoisomerase (NADP(+))	-6.079632011
NUOCD_PSEAB	NADH-quinone oxidoreductase subunit C/D	-5.786004306
DNAK_PSEAB	Chaperone protein DnaK	-5.667103455
A0A0H2ZEU8_PSEAB	Alanine/glycine:cation symporter family protein	-5.585263042
A0A0H2ZFH3_PSEAB	serine-type D-Ala-D-Ala carboxypeptidase	-5.538248955
A0A0H2ZE94_PSEAB	Alkyl hydroperoxide reductase	-5.492724516
RRAAH_PSEAB	Putative 4-hydroxy-4-methyl-2-oxoglutarate aldolase	-5.477959355
A0A0H2Z834_PSEAB	Flagellar hook-associated protein 2	-5.475610636
ARCA_PSEAE	Arginine deiminase	-5.458409552
RL4_PSEAB	Large ribosomal subunit protein uL4	-5.374887604
A0A0H2ZDT9_PSEAB	Chemotaxis protein CheV	-5.362046911
RL24_PSEAB	Large ribosomal subunit protein uL24	-5.348195756
A0A509JL21_PSEAI	Adenylate cyclase	-5.337051737
SYP_PSEAB	Proline--tRNA ligase	-5.25084362
A0A0H2ZCR1_PSEAB	R body protein RebB-like protein	-5.23645196
RL32_PSEAB	Large ribosomal subunit protein bL32	-5.148210906
A6V673_PSEP7	Porin	-5.122999336
A0A0S2TT89_PSEAI	TolC family protein	-5.117572791
MQO1_PSEAE	Probable malate:quinone oxidoreductase 1	-5.112669247
SUCD_PSEAE	Succinate--CoA ligase [ADP-forming] subunit alpha	-5.080746053
SYV_PSEAE	Valine--tRNA ligase	-5.027604472
ATPF_PSEAB	ATP synthase subunit b	-4.989355619

EFTS_PSEAB	Elongation factor Ts	-4.978803416
A0A0H2ZCQ2_PSEAB	Putative TonB-dependent receptor	-4.967871431
A0A0H2ZGG4_PSEAB	Putative exported lipoprotein	-4.953177921
A0A0H2ZKG2_PSEAB	LemA family protein	-4.943843523
A0A0H2ZIC7_PSEAB	Protein HflC	-4.923096366
A0A0H2Z6U8_PSEAB	TonB-dependent receptor	-4.86532359
LLDD_PSEAB	L-lactate dehydrogenase	-4.814039313
A0A0H2ZBY5_PSEAB	DUF883 domain-containing protein	-4.80517573
AROC_PSEAB	Chorismate synthase	-4.755118918
RS3_PSEAB	Small ribosomal subunit protein uS3	-4.659004462
A0A509JN15_PSEAI	Glucose/quinate/shikimate family membrane-bound PQQ-dependent dehydrogenase	-4.617284117
A0A3M5DNP1_PSEAI;	Ferrichrome-iron receptor	-4.614810069
SECY_PSEAE	Protein translocase subunit SecY	-4.577720346
A0A0H2Z8M2_PSEAB	Small-conductance mechanosensitive channel	-4.57341222
VFR_PSEAE	cAMP-activated global transcriptional regulator Vfr	-4.559808048
LAP_PSEAB	Aminopeptidase	-4.547764704
PYRG_PSEAB	CTP synthase	-4.492035666
A0A0H2Z999_PSEAB	Putative outer membrane receptor protein	-4.482399522
A0A6A9KBX7_PSEAI	TonB-dependent siderophore receptor	-4.465705573
OTCC_PSEAE	Ornithine carbamoyltransferase, catabolic	-4.45061828
A0A0H2ZJ18_PSEAB	Cytochrome c5	-4.406042472
A0A0H2Z9D1_PSEAB	Transcriptional regulator CysB	-4.383737076
A0A0H2ZDI1_PSEAB	Putative zinc-binding dehydrogenase	-4.350925315
PCTB_PSEAE	Methyl-accepting chemotaxis protein PctB	-4.245178071
FTSZ_PSEAE	Cell division protein FtsZ	-4.220989016
A0A0H2ZFL2_PSEAB	Putative outer membrane protein	-4.209489762
A0A0H2Z762_PSEAB	Exonuclease VII large subunit C-terminal domain-containing protein	-4.115454865
PILY1_PSEAB	Type IV pilus biogenesis factor PilY1	-4.058229298
A0A072ZIB6_PSEAI	Cytochrome c oxidase accessory protein CcoG	-4.057256562
A0A0H2ZLD6_PSEAB	Nitrite reductase	-4.021409038
SSB_PSEAE	Single-stranded DNA-binding protein	-3.989148826
A0A0H2ZG66_PSEAB	Putative iron-sulfur cluster-binding protein	-3.98879022
MURA_PSEAB	UDP-N-acetylglucosamine 1-carboxyvinyltransferase	-3.973915905
A0A0H2ZKM8_PSEAB	Putative hydroxamate-type ferrisiderophore receptor	-3.968357158
A0A0H2Z825_PSEAB	Flagellar basal-body rod protein FlgG	-3.94962816
HEM2_PSEAE	Delta-aminolevulinic acid dehydratase	-3.938687862
A0A071KU93_PSEAI	C-type cytochrome	-3.632774412
ATPD_PSEAB	ATP synthase subunit delta	-3.593188756
ETFD_PSEAE	Electron transfer flavoprotein-ubiquinone oxidoreductase	-3.540848232

DNAJ_PSEAB	Chaperone protein DnaJ	-3.481737828
A0A0H2ZIJ0_PSEAB	Carbamoyltransferase	-3.421879316
A0A0H2ZGA2_PSEAB	RND efflux membrane fusion protein	-3.313169301
FTSA_PSEAE	Cell division protein FtsA	-3.215474622
A0A0H2ZHF8_PSEAB	Putative outer membrane protein	-2.439195285
A0A367ME26_PSEAI	DUF4823 domain-containing protein	1.569201846
A0A069QEN7_PSEAI	Chromosome partitioning protein ParA	1.933965135
BAMB_PSEAE	Outer membrane protein assembly factor BamB	1.941419858
A0A0H2ZHP1_PSEAB	Paraquat-inducible protein B-like protein	2.025882742
A6V2I3_PSEA7	Lipoprotein, putative	2.027421614
A0A0H2ZDL5_PSEAB	Peptidyl-prolyl cis-trans isomerase	2.09093722
A0A0H2ZIY0_PSEAB	Dodecin family protein	4.191385679

Supplementary Table 5. List of all differentially expressed flagellar components in *ΔtamA, ΔtamB* and *ΔtamAB OM* proteome.

UniProt Id	Protein Description	LogFC *ΔtamA* vs PA14	LogFC *ΔtamB* vs PA14	LogFC *ΔtamAB* vs PA14
A0A0H2Z834_PSEAB	Flagellar hook-associated protein 2	-5.475610636	-5.475610636	-1.020982816
A0A0H2Z7W1_PSEAB	Flagellar hook protein FlgE	-3.94424859	-2.279062192	-0.383820485
FLIF_PSEAE	Flagellar M-ring protein	-3.592361808	-3.430941691	0.022923384
A0A0H2Z825_PSEAB	Flagellar basal-body rod protein FlgG	-2.794433263	-3.94962816	-0.563453483
P72129	Flagellin (Fragment)	-2.326370961	-0.384583319	-0.593177766
A0A6M5K8Z5	B-type flagellin (Fragment)	-1.464217103	-1.464217103	3.058340548
A3RJ52_PSEAI	Flagellin	-1.088882125	0.572190949	-0.243446832
FLGH_PSEAB	Flagellar L-ring protein	-0.834778256	0.733876705	0.197317268
FLGI_PSEAB	Flagellar P-ring protein	-0.049199069	-1.481075555	-0.197508165
A0A6M5KA93	B-type flagellin (Fragment)	0.905582021	3.190088506	-7.204838758

Supplementary Table 7. List of individual sample accession numbers and their corresponding names used in this study.

**Sample Name**	**Accession numbers**
Δ*tamA* 1	SAMEA118707596
Δ*tamA* 2	SAMEA118707597
Δ*tamA* 3	SAMEA118707598
Δ*tamA* 4	SAMEA118707599
Δ*tamB* 1	SAMEA118707604
Δ*tamB* 2	SAMEA118707605
Δ*tamB* 3	SAMEA118707606
Δ*tamB* 4	SAMEA118707607
Δ*tamAB* 1	SAMEA118707600
Δ*tamAB* 2	SAMEA118707601
Δ*tamAB* 3	SAMEA118707602
Δ*tamAB* 4	SAMEA118707603
PA14_WT1	SAMEA118707608
PA14_WT2	SAMEA118707609
PA14_WT3	SAMEA118707610
PA14_WT4	SAMEA118707611

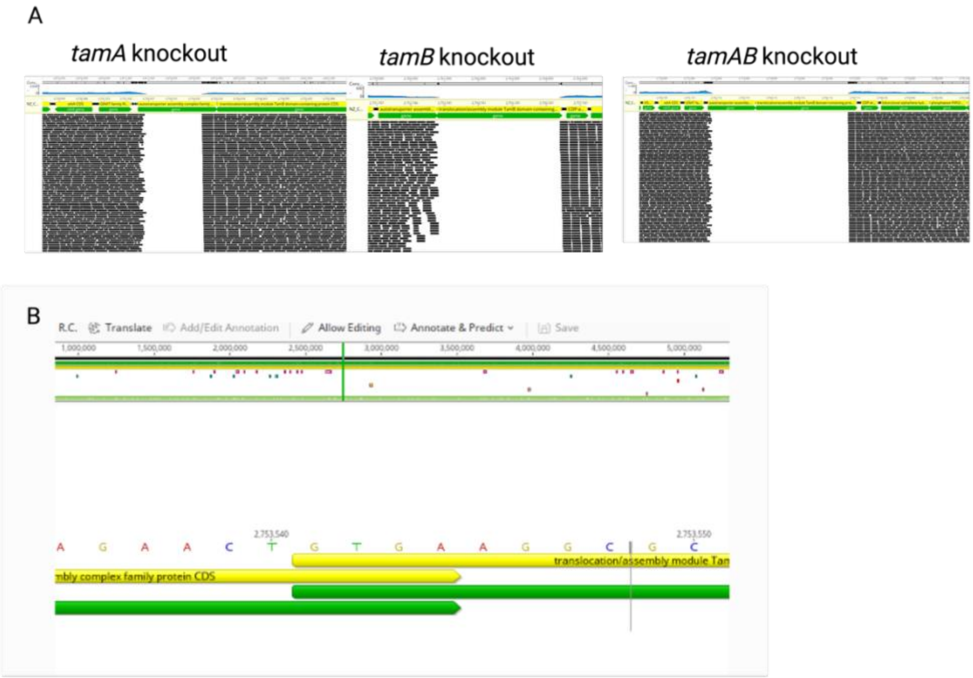

Supplementary Figure 1.
***tam* mutants and genome**
(A) reads of tam gene knockouts mapped against reference strain *P. aeruginosa PA14*
Growth curves of WT and mutant strains. OD_{600} was measured every 20 min for 24 hours using four biological replicates per strain. Curves represent raw OD_{600} values plotted over time. Growth rates reported in the text correspond to the maximal growth rate μmax (h^{-1}) calculated using Growthcurver in R
(B) overlapping stop codon of *tamA* with *tamB*

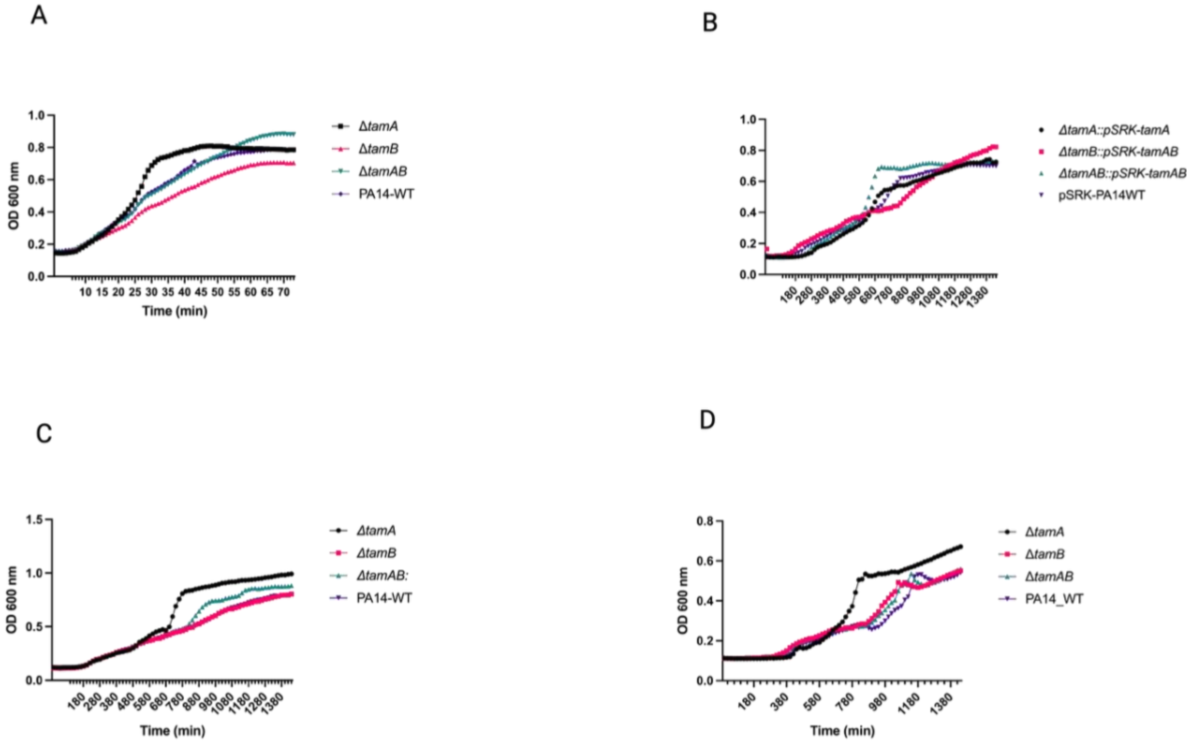

Supplementary Figure 2.

Growth profiles of tam mutants compared to the wildtype
(A) Growth curve of tetracycline sensitive mutants vs wildtype, (B) Growth curve of complemented TAM mutants vs wildtype containing pSRK background (C), Growth curve of tetracycline resistant TAM mutants compared to the wildtype showing curves represented by mean of four biological replicates. (D). Growth curve of TAM mutants in M9 media

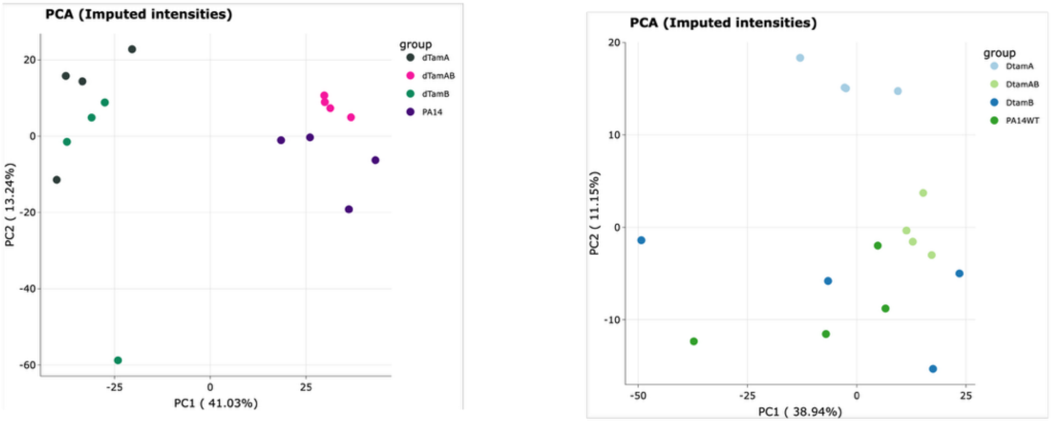

Supplementary Figure 3

Comparative principal component analysis (PCA) of OM and WCP proteome highlights tam mutants vs wildtype separation
Visualization of the overall variation in protein intensities following imputation using Principal Component Analysis (PCA). (A) PCA of outer membrane proteins (OMPs). The PC1 depicts 41.03% of the variance while PC2 shows 13.24% of the variance in total outlining 54.27% of the total variance for clustering. (B)Whole Cell proteome (WCP) PC1 and PC2 with 38.94% and 11.15% variance respectively. The PCA shows that the *ΔtamA* mutant has a distinct global protein expression pattern, separating strongly from the wildtype and other mutants, especially along PC1. *ΔtamAB* seems to show a somewhat intermediate pattern, while Δ*tamB* alone is more similar to wildtype. This suggests tamA may have a larger impact on the outer membrane proteome than *ΔtamB*.

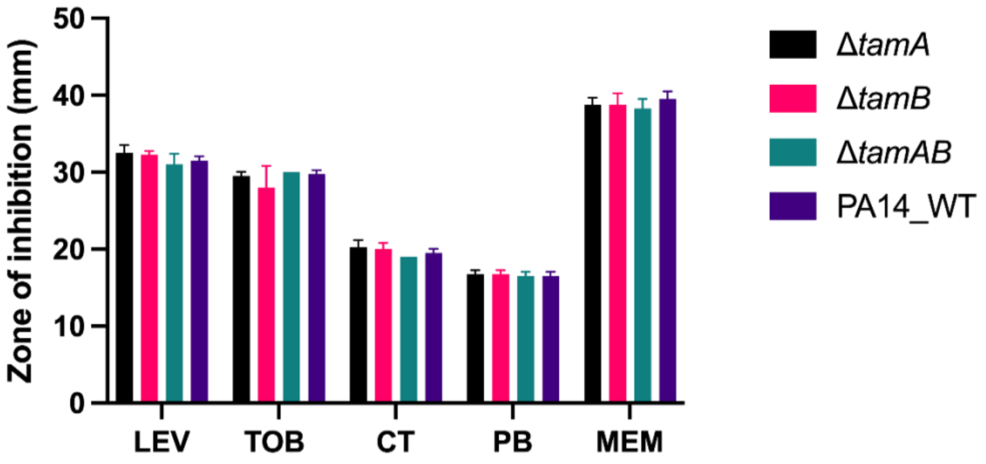

Supplementary Figure 4
***tam* mutants and wildtype exhibit comparable susceptibility to Clinically relevant antibiotics**
Antibiotics susceptibility tests for Levofloxacin (LEV), Tobramycin (TOB), Colistin (CT), PolymyxinB (PB) and meropenem (MEM) show no significant differences between mutants and wildtype. 2-way ANOVA was used statistics analysis. Nonsignificant (ns) p values not shown.

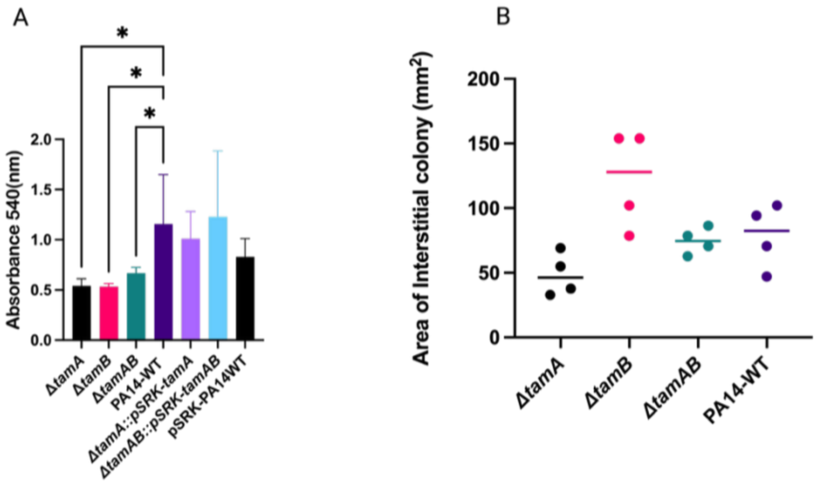

Supplementary Figure 5.

Assessment of Biofilm formation upon complementation and twitching motility of *tam* mutants compared to the WT.

(A) Complemented strains did not result in total restoration of biofilm-forming ability however there is an increase in biomass compared to the WT.

(B) Twitching of *tam* mutants and wildtype. Twitching motility was performed according to the macroscopic twitching assay of (Turnbull & Whitchurch, 2014).No significant differences observed.

Statical analysis done using One-way Anova. Complemented strains with ns p value not shown.

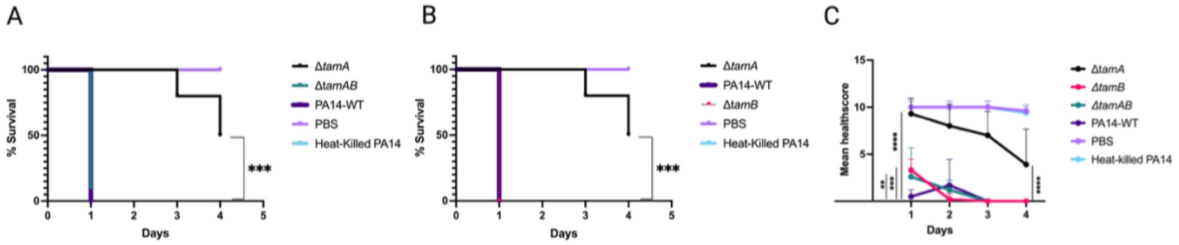

Supplementary Figure 6.

***tam* knockout is less pathogenic**

(A) Survival curve of *G. mellonella* post infection (excluding Δ*tamB* for easy visualization). The differences in the survival curves of *tam* mutants Δ*tamA* compared to Δ*tamAB* or wildtype are significant using the Mantel-Cox test with ***p <0.001.

(B) Survival curve of *G. mellonella* post infection (excluding Δ*tamAB* for easy visualization). The differences in the survival curves of Δ*tamA* compared to Δ*tamB* or wildtype are significant using the Mantel-Cox test with ***p <0.001.

(C) Health score of *G. mellonella* post infection. The health score of n=10 (where n represents technical replicates) was calculated daily for four days. Health score of all TAM mutants and the wildtype for the first day are displayed as follows; Δ*tamA* ****p<0.0001; Δ*tamB* ***p<0.0002; Δ*tamAB* **p< 0.01 shown by the black vertical lines with asterisks. Health score of Δ*tamA*-infected larvae on day 4 shown by black vertical line with asterisks ****p<0.0001. Statistical analysis obtained by Two-way ANOVA with Dunnett's post-test.

10 CFU of mutants or WT were injected and PBS and heat-killed PA14 used as controls. Larvae were incubated at 37 °C for four days, recording their survival and health score daily in accordance with the *Galleria* scoring system of Serano et al, 2023.
Figures are representative of two experiments performed independently twice.
